# Reversibly Redox-Active Iron Oxide Structures in FeNC Catalysts Identified by Microscopy and Spectroelectrochemical EPR and Mössbauer Methods

**DOI:** 10.1021/jacs.5c12396

**Published:** 2026-01-10

**Authors:** Kaltum Abdiaziz, Lingmei Ni, Derya Demirbas, Hendrik Haak, Edward Reijerse, Pascal Theis, Wulyu Jiang, Sonia Chabbra, Thomas Lunkenbein, Ulrike I. Kramm, Alexander Schnegg

**Affiliations:** 1 Max Planck Institute for Chemical Energy Conversion, Mülheim an der Ruhr 45470, Germany; 2 Catalysts and Electrocatalysts Group, Department of Chemistry, Technical University Darmstadt, Darmstadt 64287, Germany; 3 Max-Planck-Institut für Kohlenforschung, Mülheim an der Ruhr 45470, Germany; 4 28259 Fritz-Haber-Institut der Max-Planck-Gesellschaft, Berlin 14195, Germany

## Abstract

Identifying active sites in FeNC catalysts for oxygen reduction reactions (ORR) and active site changes during preparation, storage, and electrochemical cycling are key challenges in the quest for improved catalysts. In this work, high-resolution transmission electron microscopy (TEM) is combined with ^57^Fe Mössbauer and electron paramagnetic resonance (EPR) spectroscopies to investigate iron centers in high-performance FeNC catalysts with regard to their structure, coordination, and oxidation and spin states. Reversible and irreversible changes during storage, the preparation of FeNC electrodes, and their use in electrochemical cells are investigated by complementary spectroelectrochemical Mössbauer and EPR methods. Microscopy of the as-prepared FeNC materials reveals iron to be evenly distributed in isolated sites or a few atoms containing sites. Mössbauer and EPR identify weakly and strongly magnetically coupled high-spin Fe­(III) in rhombically distorted octahedral coordination or superparamagnetic clusters, high-spin Fe­(II) sixfold coordinated in iron oxides, and intermediate-spin Fe­(II) in square planar coordination. Upon oxygen exposure, a notable oxidation state change from Fe­(II) to Fe­(III) is observed, the iron is less evenly distributed, and larger iron oxide nanoparticles are formed. It is noted that for this catalyst, before and after oxygen exposure, most of the iron is bound in iron oxide structures. Under the applied potential, Fe­(III) is partially reduced to Fe­(II) in clustered and isolated or weakly coupled sites. This change is mostly reversible, suggesting structural retention of the majority of the catalyst.

## Graphical abstract

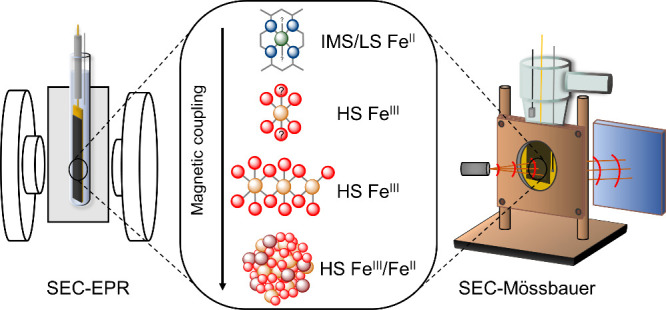

## 1 Introduction

Commercial fuel cells largely depend on platinum-based catalysts.^1,2^ However, due to platinum’s high cost and scarcity, efforts have been directed toward the design of platinum group metal (PGM) free catalysts. 3d-transition-metal nitrogen carbon catalysts (MNC) have demonstrated significant activity and peak-power density, described as maximum amount of power that can be generated per unit area or volume of a catalytic system, for oxygen reduction reaction (ORR).^3,4^ The most effective catalysts are prepared these days by independent iron, and nitrogen–carbon precursors in combination with at least one pyrolysis step.^5,6^ Although these catalysts meet the Department of Energy (DOE)^3,7,8^ targets for activity and peak-power density, their stability remains a significant challenge, as FeNCs undergo considerable degradation during operation.^9,10^ Efforts to enhance the activity and stability of FeNC electrocatalysts, therefore, require a clear identification of the active sites and a comprehensive understanding of the morphological and chemical factors affecting these sites during preparation, storage, and operation.

As a consequence of high-temperature pyrolytic preparation, a variety of iron sites are formed in FeNCs.^5,11,12^ To identify their structure and determine their relevance as catalytically active sites, X-ray absorption spectroscopy (XAS),^13,14^ ^57^Fe Mössbauer spectroscopy,^15–19^ electron paramagnetic resonance spectroscopy (EPR),^15,20–22^ and transmission electron microscopy (TEM)^23,24^ have been employed. Due to its sensitivity to iron electronic structure and coordination, ^57^Fe Mössbauer is widely used as a fingerprint method for FeNC catalysts. Room-temperature (RT) Mössbauer on FeNC has identified characteristic doublets representing different Fe environments. These doublets have been commonly referred to as D1, D2, and D3 based on their chemical shift (CS) and quadrupole splitting (Δ*E*_Q_).^11,12,25^ The predominant doublet D1 is characterized by a CS in the range 0.28–0.48 mm s^–1^ and an Δ*E*_Q_ between 0.75 and 1.23 mm s^–1^. D1 was assigned to FeN_4_ sites in the high-spin (HS, *S* = 5/2) Fe­(III) state or FeN_4_ sites in the low-spin (LS, *S* = 0) Fe­(II) state or (superparamagnetic) iron oxide clusters.^26,27^ At cryogenic temperatures, D1 partially splits up into a broad sextet component. The appearance of this fraction depends on catalyst preparation and is often clearly observed only at temperatures around 5 K or below. These sextet components were assigned to exchange-coupled Fe atoms in iron oxide nanoparticles and the remaining doublet (D1) to magnetically isolated HS Fe­(III) or LS Fe­(II) in FeN_4_ sites.^28^ While the role of these FeN_4_ moieties in ORR catalysis is highlighted, only limited information is given for the oxide species.

Doublet D2 is characterized with a similar CS to doublet D1, but Δ*E*_Q_ = 2–3 mm s^–1^. The third doublet D3 has CS = 0.8–1.2 mm s^–1^ and Δ*E*_Q_ = 1.8–2.5 mm s^–1^. Both D2 and D3 were assigned to Fe­(II)­N_4_ sites.^26,29^ To identify Fe­(III) low-spin (LS, *S* = 1/2), intermediate-spin (IMS, *S* = 3/2), and HS (*S* = 5/2) states alongside their coordination geometries in FeNC materials, Mössbauer spectroscopy was complemented by EPR spectroscopy. FeNC preparation from FeN_4_ precursors of pyrrolic character with protocols that kept the FeN_4_ macrocycle intact^15,20^ lead to axial HS Fe­(III)­N_4_ EPR spectra with characteristic turning points at *g*_eff_ = 6 and *g*_eff_ = 2 ^30,31^ Similar spectra are typically observed for square-pyramidal or octahedral HS Fe­(III) in a wide range of porphyrin complexes and heme proteins, e.g., myoglobin and hemoglobin.^32^ In this family of compounds, changes in the ligand strength can lead to HS to LS transitions that switch the EPR signal from *g*_eff_ = 6 to *g*_eff_ = 2.^33,34^

Zhao et al.^35^ recently synthesized an FeNC catalyst using individual Fe, N, and C precursors without high-temperature pyrolysis that showed a broad EPR resonance at *g*_eff_ = 6, which was assigned to Fe­(III)­N_4_. On the contrary, FeNC catalysts prepared by pyrolysis of individual Fe, N, and C precursors resulted either in very broad unstructured EPR lines or in resolved spectra with turning points at *g*_eff_ = 10, *g*_eff_ = 4.3, and *g*_eff_ = 2.^15,20–22^ These spectral features are characteristic of an octahedral coordination structure with rhombic distortion, as observed, for example, in six-fold oxygen-coordinated complexes^36,37^ or in the weakly magnetic coupled HS Fe­(III) sites in iron oxide (Fe_2_O_3_) nanoparticles in glasses^38–43^ and in zeolites.^44–46^

Iron oxide nanoparticles have been considered as degradation products in an acidic environment, which led to efforts made toward their removal.^47,48^ Our recent work showed that the removal of these and other side phases led to significant improvement of ORR activity and stability in PEMFC.^49^ In contrast, in a comparison of different benchmarking catalysts by Primbs et al.,^50^ the catalysts with the highest performance contained larger iron oxide fractions. In another work, encapsulated Fe_2_O_3_ ^51,52^ has been suggested to significantly enhance ORR activity in an acidic environment. As such, iron oxide clusters were both described as detrimental^10,17,53^ and beneficial^51,54^ for ORR catalysis over FeNC electrodes.

The controversy about the role of iron oxides might originate from a lack of detailed characterization to distinguish between different kinds and sizes. *Ex situ* characterization is usually compared with electrochemical performance, although it is known that the structural composition changes dynamically as a result of electrode preparation—and certainly also during electrocatalysis.^14,21,55^ Furthermore, the variance of the Mössbauer parameters for different FeNC materials raises doubts as to whether the active sites in the different variants of the FeNC catalysts are actually identical and whether their changes at different stages of preparation are reversible or irreversible. Finally, it remains unclear to what extent cooperativity of the iron sites is necessary to achieve a good ORR performance.

To shed light on these important questions, herein, we combined microscopy with spectroelectrochemical EPR (SEC-EPR) and SEC-Mössbauer spectroscopies to investigate iron sites in freshly prepared FeNC materials, upon exposure to air, after electrode preparation, and finally electrochemical cycling. High-resolution TEM microscopy of FeNC materials determined the distribution of Fe, C, N, and O, and their local accumulation in freshly synthesized materials, after prolonged storage and exposure to air. The characterization on the nanoscale was complemented by temperature-dependent Mössbauer and EPR spectroscopy to identify Fe-oxidation states and their coordination environment and distinguish isolated and clustered Fe sites. To assign the observed changes in the characteristic Mössbauer and EPR signatures to either irreversible structural degradation mechanisms or reversible changes in the oxidation state, SEC-EPR and SEC-Mössbauer spectroscopies (Figure 1 and Figure S1) were applied to FeNC electrodes in an inert environment and after air exposure.

**Figure 1:**
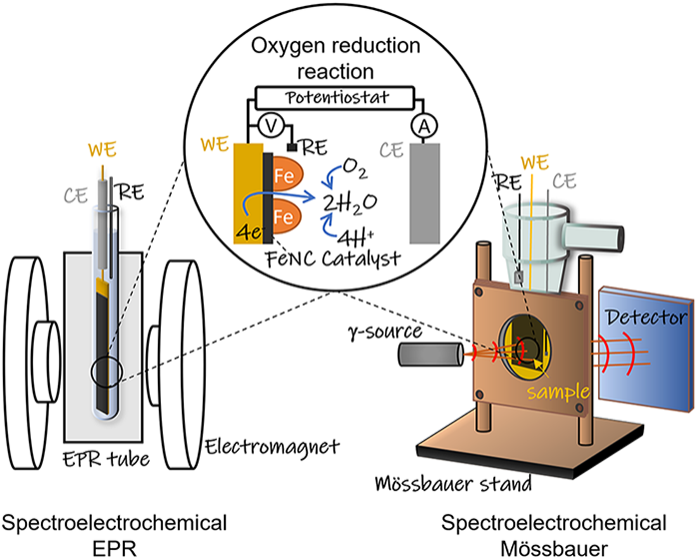
Scheme of the SEC-EPR and SEC-Mössbauer setups. For SEC-EPR, flattened gold wire (0.9 cm^2^) was used as the working electrode (WE), platinum tube as the counter electrode (CE), and silver wire as the pseudo reference electrode (RE). Inset (middle)—schematic diagram of the ORR on the WE as a possible electron transfer process. Further details, photograph and schematic diagram of the SEC-EPR setup are given in Figure S1 and Section 1 of the SI. For SEC-Mössbauer, carbon paper (5 cm^2^) was used as the WE, Ag/AgCl as the RE, and carbon paper as the CE. The WE and CE were arranged face to face inside the cell with the reference above the configuration. The electrode arrangement was fixed by copper plates outside the cell with round holes of *d* = 2.5 cm as window for the γ-rays. More details on SEC-Mössbauer can be found elsewhere.^22^

## 2 Materials and Methods

Sample preparation and experimental details of the microscopy, inductively coupled plasma emission spectroscopy (IC-OES), electrochemistry, *ex situ* EPR, and Mössbauer spectroscopy and SEC-EPR setup design are described in detail in the SI.

### 2.1 Sample Handling and Storage

FeNC materials were transferred quickly from the high-temperature oven to a sample vial under nitrogen, maintaining the inert atmosphere. The catalyst was stored in a glovebox (O_2_ < 5 ppm, H_2_O < 0.1 ppm) at ambient temperature. The FeNC materials were studied under three conditions:

- Freshly prepared in an inert atmosphere and kept under N_2_ in a glovebox (labeled **N**_**2**_). Measurements were also done in N_2_ environment.
- Preparation in an inert atmosphere and stored in a liquid N_2_ storage dewar for extended periods, labeled N_2_ long-term storage (**N**_**2**_ **LTS**). Measurements were done in a N_2_ environment.
- Exposed to air under ambient conditions (**+air**) after N_2_LTS (3 months). *Ex situ* measurements were done in an air environment, whereas the spectroelectrochemical measurements were carried out using nitrogen gas saturated electrolyte.

In the following, we refer to the storage conditions of the samples with the designations given in parentheses; catalyst powders are referred to as FeNC materials, and to electrodes prepared from these materials as FeNC electrodes.

### 2.2 SEC-EPR

The FeNC electrode for SEC-EPR was prepared by drop casting FeNC ink to give a surface coverage of 5 mg cm^–2^. Prior to electrochemical potential poising, cyclic voltammograms (CVs) of the FeNC electrodes were measured under ambient conditions. The pseudoreference was calibrated against a standard Ag/AgCl (0.3 M KCl) reference electrode; offset was found to be 0.224 V vs RHE (reversible hydrogen electrode). The same FeNC electrode was used for the entire electrochemical series, starting with 0.9, 0.75, 0.6, 0.2 V, to 0.9 V_back_. The electrode was held at the respective potential for 20 min and flash frozen in liquid nitrogen under potential application. Once frozen, the FeNC electrode in the SEC-EPR setup was transferred to the EPR spectrometer for measurements. The *in situ* experimental protocol is shown in Figure S2A.

### 2.3 SEC ^57^Fe Mössbauer

SEC-Mössbauer was performed on a spectrometer with a velocity drive equipped with a ^57^Co/Rh source (initial activity 3.5 GBq) and a proportional counter connected via a preamplifier to a CMCA-500 PC card for data acquisition. The velocity axis was calibrated against α-Fe foil. SEC-^57^Fe Mössbauer spectra were recorded with the same *in situ* cell setup (Figure 1) and *in situ* electrode preparation, as previously described.^16^ Each *in situ* electrode was prepared from a carbon paper that had an electrochemically active area of 5 cm^2^ with a loading of approximately 20 mg of FeNC material. Each electrode was labeled with consecutive numbers, e.g., electrode 1 and electrode 2, and checked subsequently for the film quality. Due to the low iron content (0.21 wt %) detected from ICP-OES in the FeNC material, always two electrodes were combined as a working electrode array for the *in situ* experiment to obtain a spectrum with good quality in an appropriate time frame. The *in situ* experimental protocol is shown in Figure S2B. For example, in a first run, electrode 3 and electrode 4 (E3E4) were combined for a test in which the *in situ* experiment was started with potentials of 0.9, 0.75, 0.6, 0.2 V, and 0.9 V_back_, by holding each potential separately for about 3 h (Figure S2B). Afterward, a potentiostatic hold (PSH) *in situ* test was performed at a specific potential of 0.9 V for approximately 7 h to complete the experiment. Similarly, the second test was performed with E2E5, the third test with E6E7 and the fourth test with E8E9. The protocols for the additional three tests were similar, but the final PSH was made at 0.75, 0.6, and 0.2 V. As indicated by the star in Figure S2B, CV measurements were conducted on each electrode by the same protocol used under standard rotating ring disk electrode (RRDE) N_2_ conditions before the potential hold at 0.9 V and after the potential hold at 0.9 *V*_back_. For data analysis, all *in situ* Mössbauer spectra measured at the same potential were summed up.

## 3 Results

### 3.1 Electrochemical Performance

Cyclic voltammetry and the ORR performance of the FeNC catalysts were investigated using an RRDE experiment at room temperature, with a catalyst loading of 0.51 mg cm^–2^. As visible from the CV in Figure S3A, the redox potential for the Fe­(III)/Fe­(II) transition was at 0.64 V. The linear sweep voltammetry (LSV) curve under O_2_ saturation depicted in Figure S3B revealed an onset potential of 0.85 V, a half-wave potential of 0.74 V, and a mass-related kinetic current density of 1.3 A g^–1^ at 0.8 V, which is in the same range as benchmarking FeNC catalysts.^50^ Moreover, the maximum H_2_O_2_ yield (Figure S3C) at the beginning of the test (BoT) was only 6.3%, whereas it is noted that for precise yields, measurements at lower loadings should be performed. The average number of transferred electrons from the Levich–Koutecky analysis was 3.92 (at 0.6 V, Figure S3D), indicating that the ORR for this FeNC material was a near four-electron transfer reaction. Still, in comparison to other FeNC material, a good selectivity toward the four-electron reduction was obtained. For example, the Fe_0.5_ catalyst has a H_2_O_2_ yield of 4.2% at 0.4 mg cm^–2^.^53^ To further see to what extent the FeNC material got deactivated by the *in situ* conditions, one of the protocols for the *in situ* Mössbauer conditions (with the last step at 0.6 V, Figure S3C), data at end of test (EoT) was applied on an RDE with a standard catalyst loading (0.51 mg cm^–2^). A slight decrease in activity was observed, with the half-wave potential reduced by 13 mV and hydrogen peroxide increasing to 7.5%, the electron transfer number remains the same. Hence, a slight degradation can be observed due to the *in situ* Mössbauer protocol. CV and chronoamperometry traces of each set of electrodes subjected to *in situ* Mössbauer is shown in Figures S4 and S5 and for SEC-EPR in Figure S6. All electrochemically determined values are summarized in Table S1.

### 3.2 EPR and Mössbauer spectroscopy of FeNC Materials

EPR spectroscopy is highly sensitive to half-integer Fe spin states, allowing in particular the assignment of the spin state and coordination geometry of Fe­(III) through line-shape analysis.^56^ The RT X-band CW EPR spectrum of FeNC materials after long-term storage in N_2_ (N_2_LTS) exhibited a broad EPR resonance centered at an effective *g*-value *g*_eff_ = 2.1 and a peak-to-peak line width Δ*H*_p–p_ ≈ 160 and 185 mT at 298 K (Figure 2A) and 100 K (Figure 2B), respectively. This broad EPR signal is assigned to exchange-coupled iron in superparamagnetic iron oxide structures.^39^ After air exposure (+air), the EPR intensity increased by a factor of 1.3, while the resonance position and the line width remained nearly the same.

**Figure 2:**
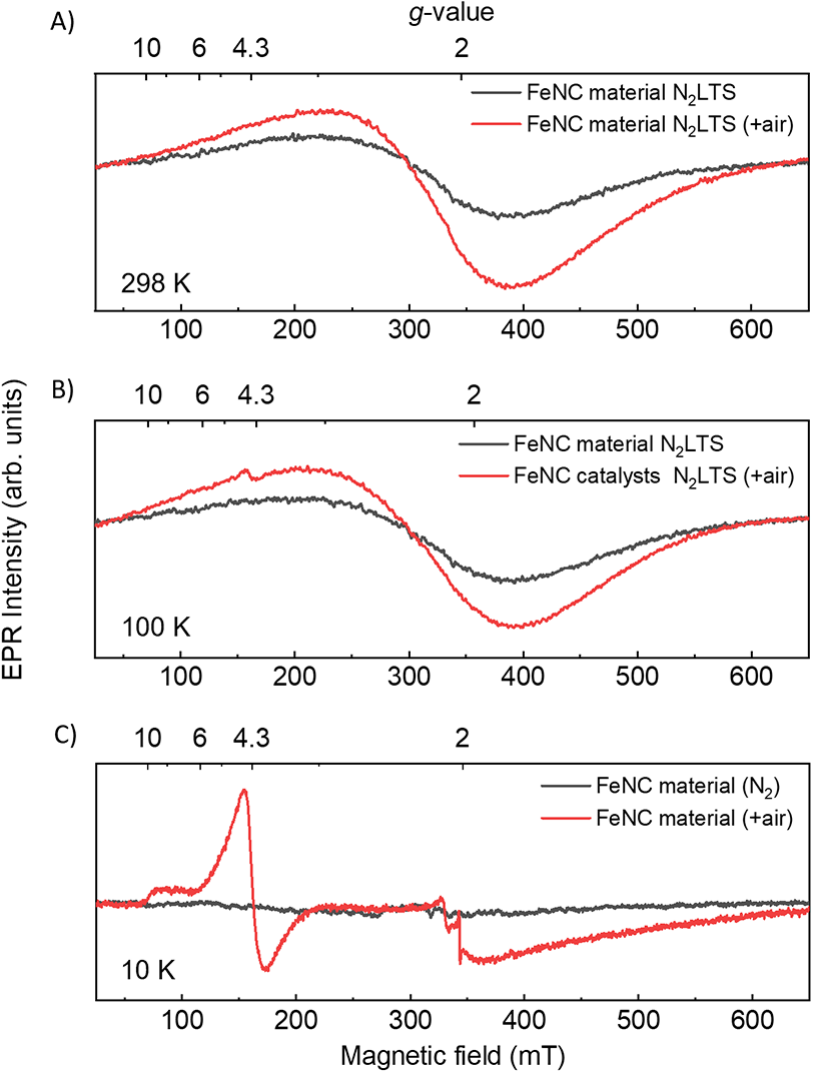
X-band CW EPR spectra of FeNC materials measured at (A) RT and (B) 100 K after long-term storage in N_2_ (N_2_LTS, black traces) and after air exposure (air, red traces). (C) X-band CW spectra of FeNC materials measured at 10 K under N_2_ (black trace) and +air conditions (red trace).

Upon lowering the temperature to 30 K, the line width Δ*H*_p–p_ continuously further increased (Figure S7) until it was indistinguishable from the baseline at 10 K. Such behavior is typical for superparamagnetic iron structures.^39,57^ Lowering the temperature to 100 K, in addition, gave rise to a small EPR peak at the characteristic resonance position *g*_eff_ = 4.3 in the FeNC materials measured under +air conditions (Figure 2B, red trace). This signal (*g*_eff_ = 4.3) was superimposed on the broad EPR line and contributed to less than 1% of the overall EPR intensity. At even lower temperatures of 10 K, this contribution, with a low field edge at *g*_eff_ = 10 (75 mT at X-band conditions), a maximum at *g*_eff_ = 4.3 (160 mT), and a broad tailing contribution that extends beyond *g*_eff_ = 1 (340–750 mT), was dominating the EPR spectrum of air-exposed FeNC materials (Figure 2C, red trace). The signal at *g*_eff_ = 4.3 is a common feature for HS Fe­(III) (*S* = 5/2) with strong rhombicity in the zero-field splitting (ZFS), *E*/*D* ∼ 1/3, where *D* and *E* are the axial and rhombic ZFS parameters, respectively. Similar EPR spectra have been reported in other FeNC material (Fe_0.5_).^15,20–22^ In FeNC samples that had not been exposed to air, the *g*_eff_ = 4.3 component was missing at all measured temperatures down to 10 K (Figure 2C, black trace). The *g*_eff_ = 10, *g*_eff_ = 4.3, and *g*_eff_ = 2 components of the 10 K (+air) EPR spectra exhibited the same MW power dependence (Figure S9). This suggests that they have similar spin relaxation times. Additional CW Q-band experiments resulted in drastically altered EPR spectra (Figure S8B, black line), with the maximum of the spectrum shifted from *g*_eff_ = 4.3 to *g*_eff_ = 2. This change in line shape upon increasing the mW-excitation energy from 0.33 cm^–1^ (X-band) to 1 cm^–1^ (Q-band) indicates that the axial ZFS is less than the Q-band mw energy (*D* < *h*ν_Q‑band_ ∼ 1 cm^–1^).^42,58,59^ The large rhombicity and the relatively small ZFS of the observed HS Fe­(III) differ from the EPR properties typically observed for HS Fe­(III) in FeN_4_ macrocycles, where four equatorial nitrogen ligands lead to large axial ZFS and hence a characteristic EPR peak around *g*_eff_ = 6.^15,30,31^

Decent fit between experimental and simulated EPR spectra (Figure S8, Section 3 in the SI) was only obtained, assuming strongly distributed ZFS parameters (ZFS-strain). Large ZFS-strain indicates significant site-to-site disorder in the Fe coordination and implies that HS Fe­(III) is not present in a single well-defined coordination but rather in a large distribution of isolated and weakly magnetically coupled structures. This finding aligns with the high degree of amorphization of the FeNC material as a result of the high-temperature pyrolysis.

^57^Fe Mössbauer in the temperature range from 80 to 1.5 K was applied to FeNC materials under N_2_, N_2_LTS, and +air conditions (Figure 3). The same three doublets were observed in 298 K and 80 K Mössbauer spectra (Figure S10) with line shape typically encountered in FeNC materials. The increase in D1 and decrease in D3 from 80 to 298 K are attributed to partial sample oxidation during sample transfer between measurements. This spectrum was dominated by a doublet D1, and one or two additional doublets, which are often referred to as D2 and D3, respectively. In the present case, the following Mössbauer doublets have been fitted to the spectra in Figure 3: D1 [CS = 0.4 mm s^–1^, Δ*E*_Q_ = 1.0 mm s^–1^], D2 [CS = 0.4 mm s^–1^, Δ*E*_Q_ = 2.5 mm s^–1^], and D3 [CS = 1.1 mm s^–1^, Δ*E*_Q_ = 3.0 mm s^–1^] at 80 K in N_2_ environment (Figure 3A). The Δ*E*_Q_ and CS values for D1–D3 agree well with similar FeNC materials.^17–19,21^ Based on their Δ*E*_Q_ and CS values, D1 could either originate from HS Fe­(III) or an LS Fe­(II), D2 from IMS Fe­(II), and D3 from HS Fe­(II).^29,60^

**Figure 3:**
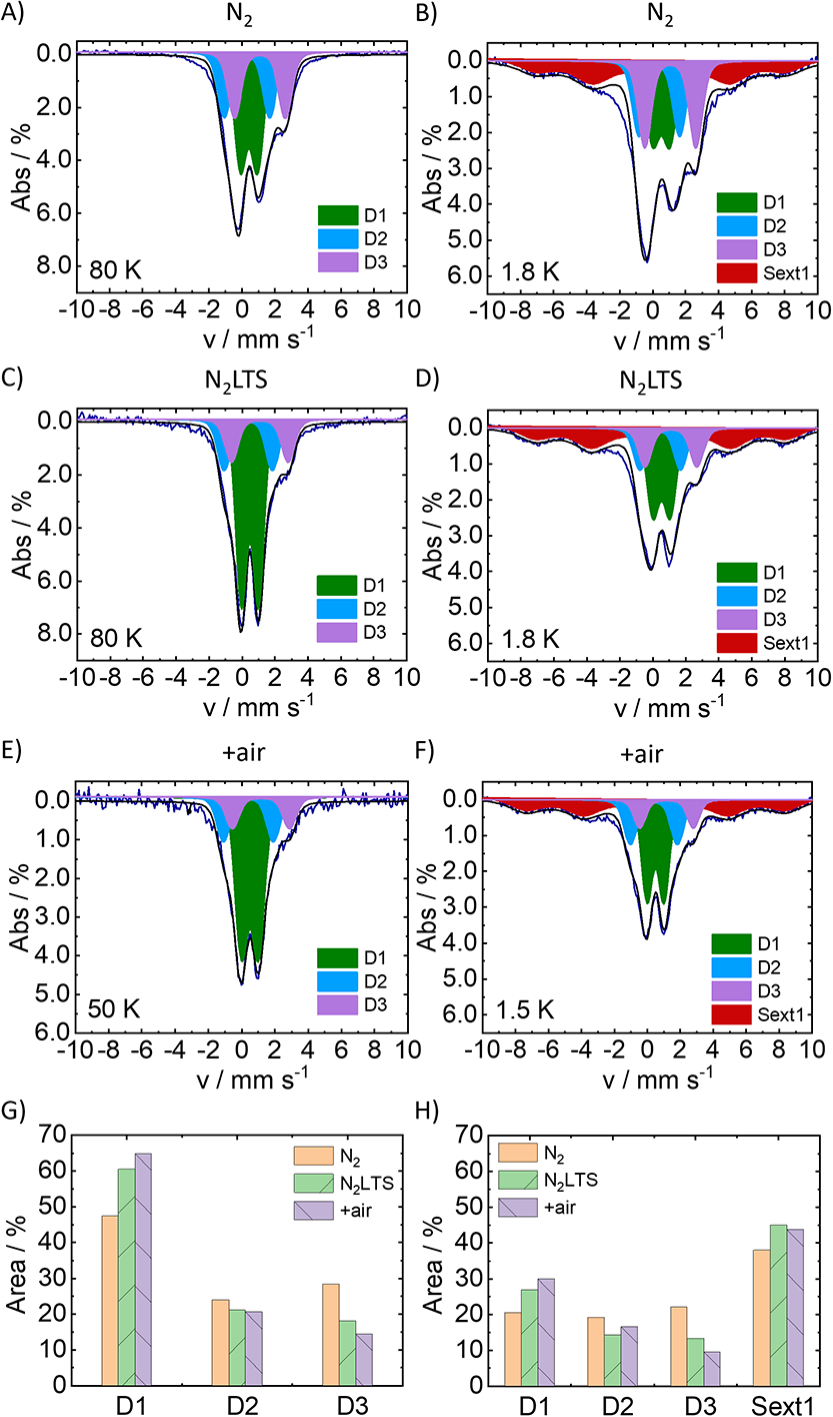
^57^Fe Mössbauer of FeNC materials under N_2_, N_2_LTS, and + air conditions. Freshly, prepared FeNC materials under N_2_ measured at (A) 80 K and (B) 1.8 K, FeNC materials under N_2_LTS conditions measured at (C) 80 K and (D) 1.8 K, and FeNC + air conditions at (E) 50 K and (F) 1.5 K. Experimental spectra are plotted alongside simulations. Bar graphs indicate the different Mössbauer features fitted to spectra at (G) 80/50 K and (H) 1.8/1.5 K. The given percentage of absorption area of the individual components in (G) and (H) represents their percentage of the total Fe amount. Mössbauer spectra were fitted using Fit model 2; details about the simulation model can be found in Section 4 and Table S3 of the SI.

Lowering the temperature below 2 K resulted in the appearance of sextets (Figure 3B). The sextets were not visible in the 80/50 K Mössbauer spectrum and started splitting from D1 at *T* = 5 K (Figure S11), consistent with the literature .^17,28^ The sextet based on its Mössbauer parameters and line broadening was assigned to partially relaxed iron oxide AC or NPs (CS = 0.45 mm s^–1^, 48 T). The appearance and relative increase of the sextet contribution at 1.5 K was accompanied by a pronounced decrease of D1 (Figure S3H). The 1.5 K Mössbauer spectrum could be again very well simulated assuming the doublets D1, D2, and D3 and partly relaxed sextets (Figure 3F). It should, however, be noted that the contributions derived from these simulations are influenced by the simulation model (see details in Section S4 in the SI). The sextet splitting occurring at very low temperatures indicates small iron oxide AC^61,62^ or NPs significantly contributed to D1. Due to the fact that the sextets may not have been fully relaxed even under 2 K, the true hyperfine splitting of the sextet signal could only be estimated, while a clear-cut assignment to a particular iron structure based on the sextet splitting is hampered by the poor resolution of the sextet peaks.

The respective contributions (doublets and sextets) to the Mössbauer spectrum have strikingly broad line widths. The reason for broadened Mössbauer lines may be either short relaxation times (Lorentzian line shape) or a strong distribution of the static CS and Δ*E*_Q_ parameters (Gaussian). Since pronounced site-to-site disorder of the coordination environment was observed in the EPR spectra of FeNC materials (Figure 2C), and considering the sensitivity of the Mössbauer parameters to the coordination environment, a Voigt line, a convolution of Gaussian and Lorentzian lines, with contributions 0.2–0.3 mm s^–1^ (Lorentzian) and 0.65–0.8 mm s^–1^ (Gaussian) were assumed in the simulations of the Mössbauer spectra (see SI Section 4 for more detail). Also, based on a similar Lorentzian line width (0.3 mm s^–1^) for well-defined Fe phthalocyanines^63^ and the very pronounced site-to-site disorder assigned in the EPR simulations, site-to-site disorder in the Fe coordination is also assigned as the dominating Gaussian line broadening mechanism in the studied FeNC.

Mössbauer spectra of FeNC material in N_2_, N_2_LTS, and after air exposure were simulated assuming the same doublets and sextets in the spectra, however with different relative intensities. FeNC material in its as-prepared conditions (N_2_) exhibited a majority D1 (47%) with similar amounts of D2 (24%) and D3 (28%). Upon storage (N_2_LTS) and air exposure (+air), D1 increased by a factor of 1.3 and 1.4 from the as-prepared sample (N_2_), respectively. D2 stayed relatively the same, but D3 decreased (Figure 3G), which indicated a partial conversion of D3 to D1 and a structural relationship between these two sites. A similar trend was observed at 1.8 and 1.5 K, with an increase in both D1 and sextets (Figure 3H). Comparison of the 1.8 K Mössbauer spectra of as-prepared catalysts (Figure 3B) and after exposure to air (Figure 3F) shows an increase of D1 (and sextet), which is accompanied by an increase of the RT broad EPR signal. Octahedral HS Fe­(III) with different magnetic coupling strengths are identified as the main causes of both D1 and sextets in the Mössbauer spectra and of the broad and narrow EPR components of the RT and low-temperature EPR spectra. In both spectroscopies, however, the relaxation properties strongly influence the line shapes and the relative contribution of the respective components to the spectra. Therefore, it is not straightforward to assign the respective EPR and Mössbauer components to each other. These findings clearly indicate the formation of additional exchange-coupled paramagnetic iron sites, presumably iron oxides, upon long-term storage and air exposure.

### 3.3 Microscopy of FeNC Materials

To investigate the distribution of isolated and clustered iron structures in the carbon matrix, microscopy images were taken on FeNC materials stored in N_2_ and exposed to air. High angle annular dark-field (HAADF), scanning transmission electron microscopy (STEM) coupled with energy-dispersive X-ray spectroscopy (STEM-EDX) (Figure 4) showed that the main elements present in the investigated FeNC materials were carbon (C), nitrogen (N), oxygen (O), and iron (Fe). In as-prepared FeNC materials (N_2_, Figure 4A), EDX mapping revealed that Fe, O, and N are homogeneously distributed within the carbon framework. No dense iron particles were observed. The presence of these elements was also confirmed by EDX spectra (Figure S12) after N_2_ treatment. Upon contact with air, some areas showed an accumulation of iron and oxygen, indicating the formation of iron oxide nanoparticles (NPs) (Figure 4B). The formation of iron oxide NPs was also observed in FeNC ink samples with sulfonated tetrafluoroethylene-based fluoropolymer copolymer (Nafion) as a conductive polymer binder (Figure S13).

**Figure 4:**
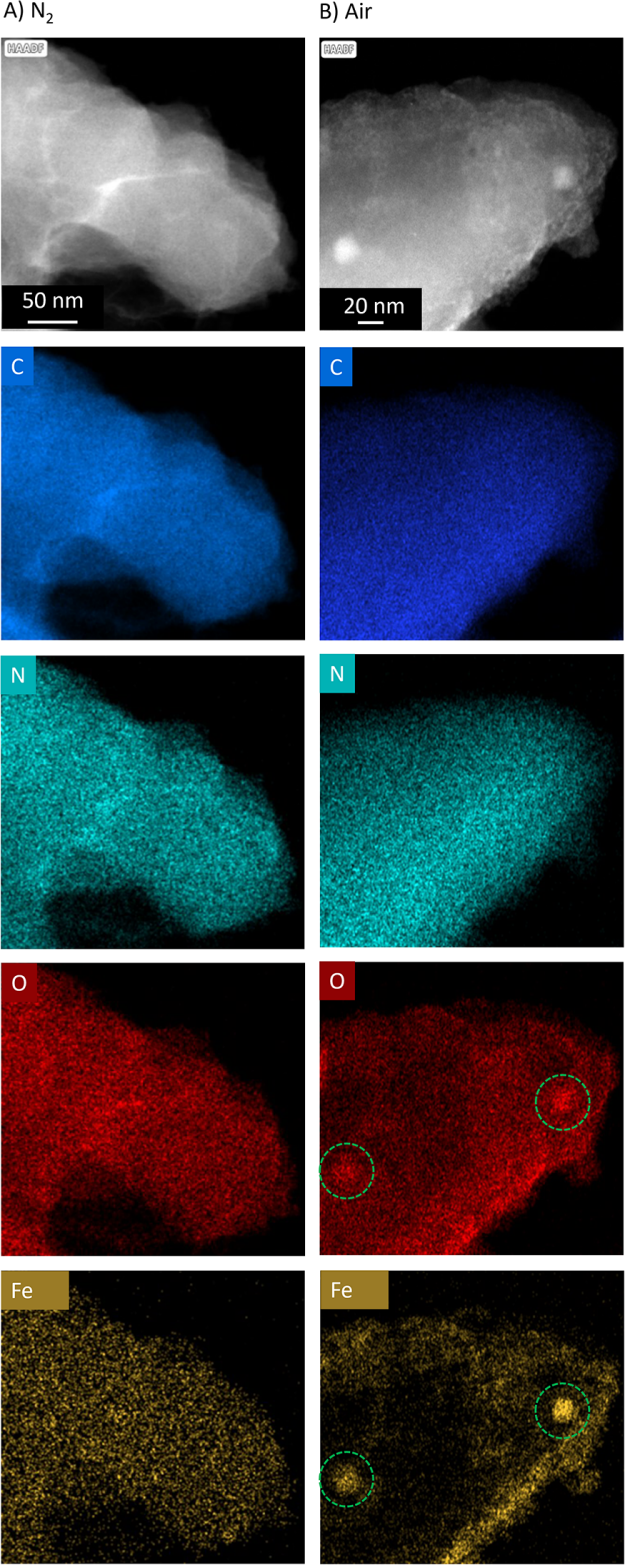
STEM images with EDX elemental mapping of FeNC materials measured under (A) N_2_ and (B) +air conditions. From top to bottom are shown HAADF-STEM and STEM-EDX images of carbon (blue), nitrogen (cyan), oxygen (red), and iron (yellow). Dashed circles show iron oxide nanoparticles.

The Fe distribution at the atomic scale was further investigated by high-resolution STEM (HRSTEM). The yellow circles in Figure 5 show the dispersion of isolated Fe atoms across the carbon surface in N_2_ (Figure 5A) and +air (Figure 5B) conditions. A reduction in the number of uniformly distributed iron sites was observed in the regions examined when the FeNC material was exposed to air. Quantitative statistical analysis^64^ of the decrease in uniformly distributed Fe sites is challenging due to sample thickness; however, this decrease is consistently observed across multiple regions of the FeNC catalyst (Figure S14). Fe clusters, containing few Fe atoms in close proximity (<0.5 nm, blue dashed circles, Figure 5C), were also found alongside single Fe atoms. In summary, microscopy revealed that iron is distributed in the carbon framework, either as single atoms or sub-nm-sized structures of few iron atoms, as well as larger iron oxide nanoparticles. The latter predominantly appear after air exposure of the FeNC material. In the following, we refer to these situations as isolated Fe, atomic clusters (AC) of iron, and iron oxide nanoparticles (NPs).

**Figure 5:**
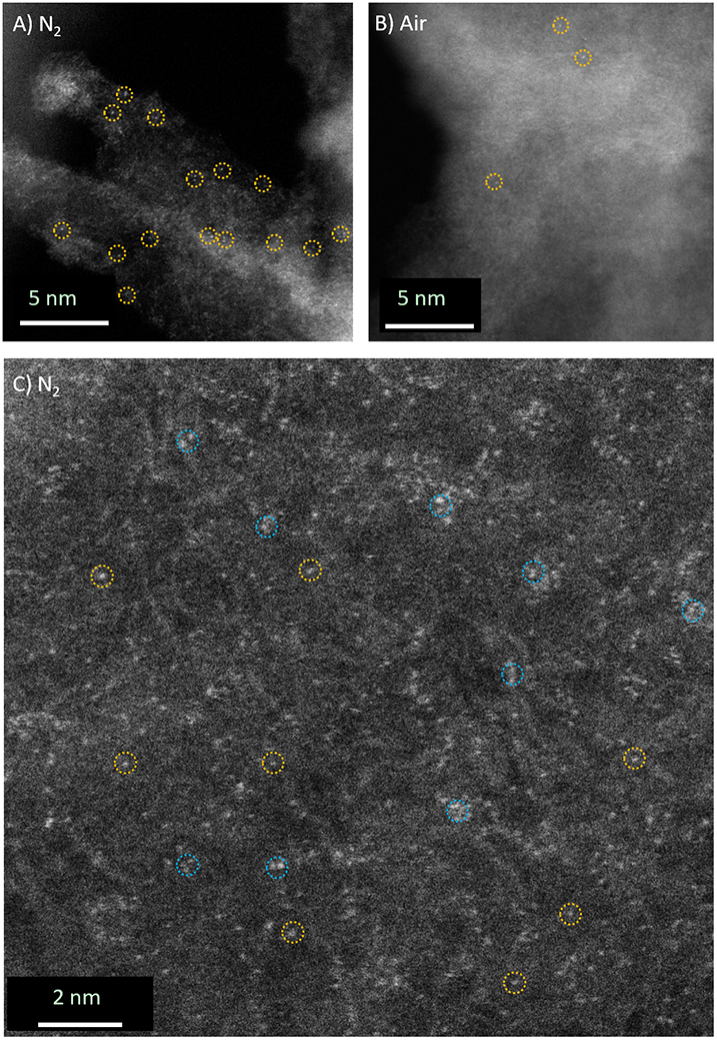
ADF-HRSTEM images of FeNC material (A) under N_2_ and (B) +air conditions. (C) HRSTEM image of FeNC under N_2_. Encircled are regions (circle diameter 0.5 nm) where a single Fe (yellow dashed circle) or more than one Fe (blue dashed circle) was identified.

### 3.4 Spectroelectrochemical EPR and Mössbauer Spectroscopy

To elucidate which iron structures in FeNC undergo irreversible changes upon degradation and air exposure and which iron sites can be electrochemically cycled between different oxidation states or coordination environments, SEC-EPR and SEC-Mössbauer spectroscopies were set up and applied.

To investigate the potential-induced structural changes in FeNC electrodes, the as-prepared catalyst in N_2_ environment was compared with the material after exposure to air under noncatalytic conditions (N_2_-saturated electrolyte). The focus was on characterizing and monitoring the electroreduction of iron sites in FeNC catalysts without interference from substrate binding but not under ORR conditions. SEC-EPR of the as-prepared FeNC electrode (N_2_) at 10 K under inert conditions revealed no signal before electrochemical treatment (Figure 6A, black line). At 0.9 V (Figure 6A, orange line), an EPR signature appeared that resembled the Fe­(III) EPR spectrum of the air-exposed FeNC material.

**Figure 6:**
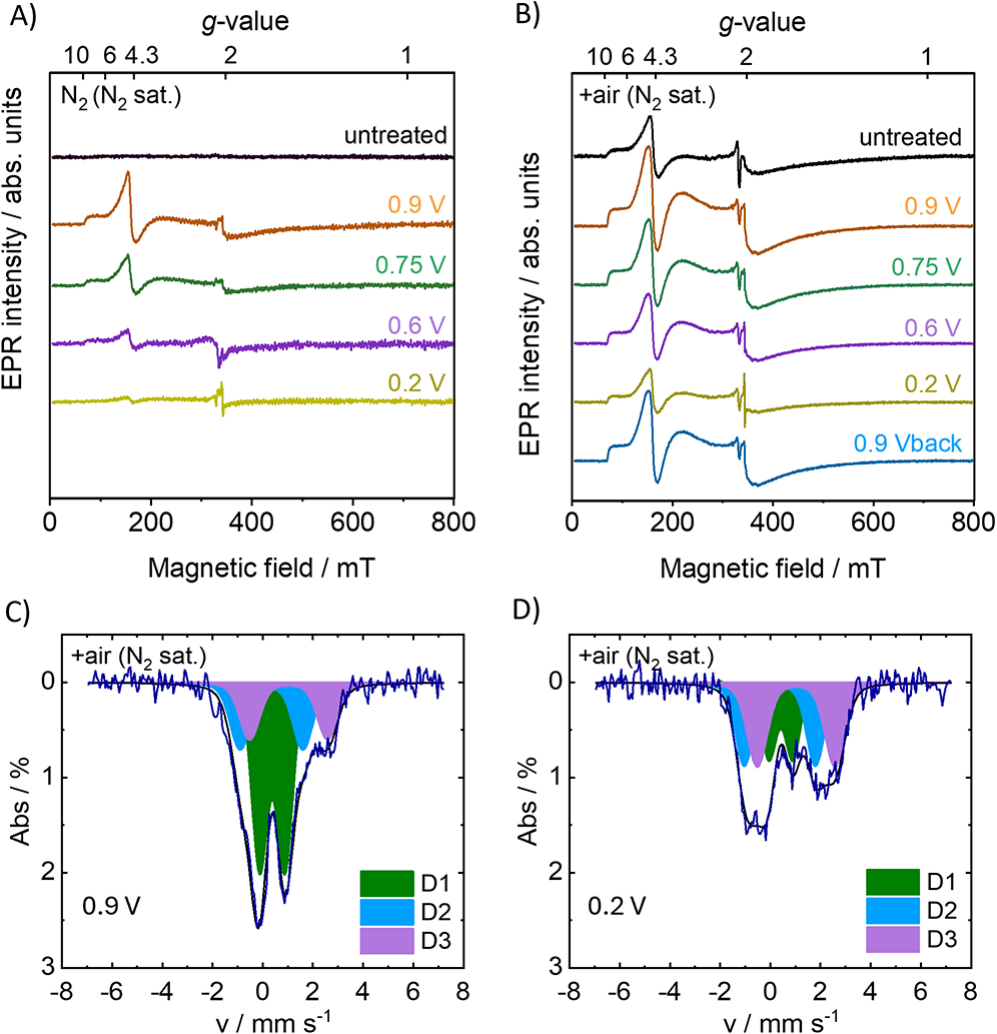
SEC X-band CW EPR (*T* = 10 K) of (A) N_2_ FeNC electrode and (B) +air FeNC electrode both measured in N_2_-saturated electrolyte at 0.9 V (orange), 0.75 V (green), 0.6 V (purple), 0.2 V (yellow), and 0.9 V_back_ (blue, only for +air). RT SEC-Mössbauer spectra of +air FeNC electrodes in N_2_-saturated electrolyte at potentials of (C) 0.9 V and (D) 0.2 V experimental Mössbauer spectra (blue) and simulations (black) with Fit Model 2 assuming doublets D1 (green), D2 (blue), and D3 (purple).

Stepwise reduced electrochemical potentials of 0.75, 0.6, and 0.2 V led to a decrease in EPR signal intensity. A similar modulation of the EPR signal upon application of a potential was observed for the FeNC material exposed to air (Figure 6B), prior to the electrochemical cycling. Also here, the signal intensity decreased as the potential was reduced from 0.9 to 0.2 V. Unlike FeNC (N_2_), where the signal nearly vanished at 0.2 V, the same EPR signal with reduced intensity was already present before the application of a potential and did not completely disappear after applying 0.2 V for +air (Figure 6A,B, yellow line). This underlines that *ex situ* oxidized Fe was formed in +air that could differ from the environments formed upon the oxidation that occurred at 0.9 V for the initially inert electrode N_2_, likely being associated with the iron oxides. Nonetheless, the influence of the potential on EPR signal intensity was consistent across both conditions. After reoxidizing the FeNC electrode back to 0.9 V following electrochemical cycling, the EPR signal intensity was nearly restored, showing only a 12% decrease compared to the initial intensity at 0.9 V (Figure 6A). This observation suggests that the change in the EPR signal was largely due to a reversible reduction of Fe­(III) and only minor to an irreversible structural change, indicating a possible missing electrochemical connection.

Furthermore, SEC-Mössbauer was performed at the same potentials as the SEC-EPR experiments to follow the electrochemical conversion *in situ* and to identify both the EPR-active Fe­(III) and EPR-silent Fe states (Figure 6C,D and Figure S14). The *in situ* SEC-Mössbauer was again simulated with Fit Model 2 assuming three doublets, D1, D2, and D3. Like in the FeNC materials at 298 and 80 K, no contribution of sextets was observed at room temperature. SEC-Mössbauer spectra of FeNC electrode (+air) at 0.9 V were measured in N_2_-presaturated electrolyte, during which N_2_ flowed above the electrolyte. The 0.9 V SEC-Mössbauer spectrum shown in Figure 6C resembled those of air-exposed FeNC materials (Figure 3E). The overlay and fitted SEC-Mössbauer spectra under *in situ* conditions 0.75, 0.6, and 0.9 V_back_ as well as the Mössbauer fit parameters are presented in Figure S15 and Table S4, respectively. We note that the measured Mössbauer intensity decreased during the electrochemical experiments. We attribute the decrease in total counts per time interval to partial leaching of iron from the FeNC electrode into the liquid solution, where it was no longer contributing to the Mössbauer signal. In connection with a possible demetalation of FeN_4_ sites, it should be noted that EPR did not detect large amounts of radicals in the catalyst (that would show up as a sharp signal at *g*_eff_ = 2), which would have been expected for a significant amount of demetallized structures.

In order to separate the contributions of leaching from the potential-induced changes of the individual doublets in the Mössbauer spectrum, the contribution of leaching was determined independently during the individual potential steps. We found that leaching within the accuracy of the experiment involves D1. The leaching is mainly observed during the first potential cycle (Figure S16) between BoT and EoT, in which an average of 19 ± 3% of the iron is leaving the catalyst (Figure S17E and Table S5). The loss is most pronounced within first potential cycle from 0.75 to 0.2 V (Figure S18 and Table S6). This is consistent with independent observations on similar materials.^65^ Once the potential cycle has been completed, it is no longer detectable within the detection limit of the SEC-MS spectra of approximately 5% (Figure S18D). Figure 7 shows the change in the EPR signal intensity for the different potential steps alongside potential-induced changes in D1, D2, and D3 obtained from simulations with Fit Model 2 to the SEC-Mössbauer spectra shown in Figure 6 and Figure S15.

**Figure 7:**
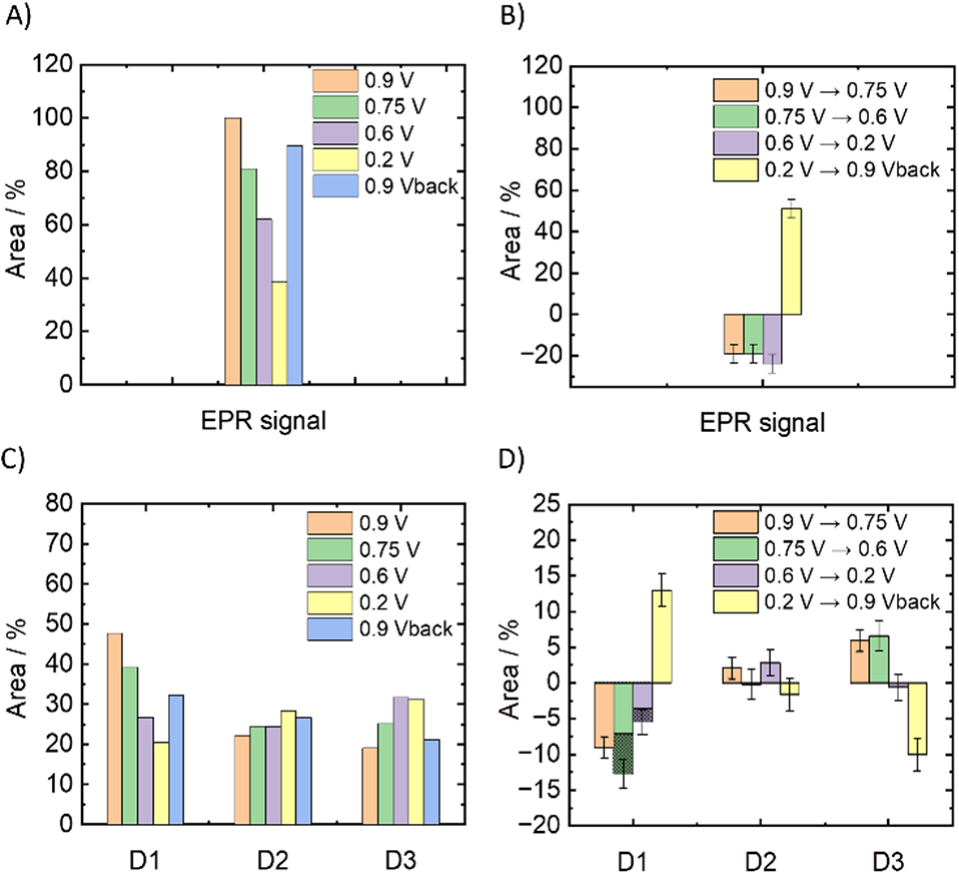
Bar graphs of (A) the potential-dependent integrated areas of the entire EPR spectrum at 0.9 V (orange), 0.75 V (green), 0.6 V (purple), 0.2 V (yellow), and 0.9 V_back_ (blue), (B) changes of the integrated EPR signal between the given potential steps, (C) the potential-dependent integrated areas of doublets D1, D2, and D3 normalized to the total area of the Mössbauer spectrum at 0.9 V BoT, and (D) changes of the integrated doublet contributions between the given potential steps. In (D) is given the leaching contribution (shaded) in D1 for the given potential steps, as determined in Section 6 in the SI. Note, in the RT Mössbauer spectra, leaching of iron species from the solid-state catalyst into the electrolyte removes the contribution of this species from the spectrum, while in frozen state, EPR ferric iron in both the catalyst and the electrolyte contributes to the spectrum.

As the potential was gradually reduced from 0.9 to 0.2 V, the integrated area of D1 relative to the initial spectrum (0.9 V BoT) decreased by −9% (0.9 V → 0.75 V), −13% (0.75 V → 0.6 V), and −6% (0.6 V → 0.2 V). Changing the potential back to 0.9 V increased D1 by 13% (0.2 V → 0.9 V back). Leaching affected D1 by 7% and 3% at potential steps of 0.75 V → 0.6 and 0.6 V → 0.2 V, respectively (Figure 7D, shaded). This means that 24 ± 3% of the initial D1 is lost through leaching, 34 ± 3% shows no changes in the SEC experiments, and 28 ± 3% undergoes a reversible redox cycle. In addition, 14% of the redox induced changes in D1 are not recovered. This is similar to 11% of unrecovered HS Fe­(II) in SEC-EPR.

Within the uncertainty of the experiment (∼5%), D2 shows no or only a slight positive change when the potential is changed from 0.9 to 0.2 V. D3 increases by 6% (0.9 → 0.75 V) and 7% (0.75 → 0.6 V) between 0.9 and 0.6 V. No change is observed between 0.6 and 0.2 V. Between 0.2 V and back to 0.9 V, a significant decrease of −11% can be observed, which is approximately anti-proportional to the increase of D1 at this potential step.

## 4 Discussion

Below, key experimental findings are discussed in relation to selected previous spectroelectrochemical studies on FeNC materials and electrodes in acidic environments, with a focus on SEC-Mössbauer. For FeNC materials studied herein, microscopy of as-prepared FeNC materials showed the presence of iron sites containing isolated iron or few iron atoms containing clusters (AC) and larger iron oxide nanoparticles (NPs). Most of the iron was found to be distributed in the investigated regions of the prepared FeNC materials. Despite that oxygen was found to be present in the material prepared under inert conditions, air exposure led to less distributed iron and the formation of NPs that had not been observed in the as-prepared materials (N_2_). This indicated the migration of Fe from isolated and weakly magnetically coupled sites to AC and NPs. FeNC materials stored under liquid nitrogen exhibited a very broad RT EPR spectrum centered at *g*_eff_ = 2, indicating exchange-coupled iron (presumably HS Fe­(III)) in superparamagnetic iron oxide structures (AC or NPs). Prior to contact with air, no EPR signal was detected at 10 K. Under these conditions, iron was either EPR-silent (e.g., Fe­(II) or Fe­(IV)) or present in superparamagnetic structures, with such broad EPR lines that they were not detectable with CW EPR. Air exposure led to an increase in the broad superparamagnetic EPR signal and to an additional smaller EPR contribution with a maximum at *g*_eff_ = 4.3. At 10 K, where the line width of the superparamagnetic contribution exceeds the observation window of the X-band CW EPR, this contribution dominated the EPR spectrum of the air-exposed FeNC materials. The *g*_eff_ = 4.3 signal was assigned to isolated or weakly coupled rhombic HS Fe­(III) in the disordered regions of the iron oxide structures. Neither before nor after air exposure, a significant EPR contribution around *g*_eff_ = 6, characteristic for HS FeN_4_ macrocycles with square-pyramidal or octahedral coordination, was detected.

Pyrolyzed materials often show sharp EPR signals at *g*_eff_ = 2, originating from defects (e.g., carbon dangling bonds) in the carbon matrix.^66^ The latter are not observed in this study, which indicates a low number of defects in the carbon matrix. In this context, it needs to be noted that FeNCs can be synthesized by many different pathways, which can lead to the formation of various Fe aggregates, including carbides and sulfides, which are expected to lead to distinct EPR signatures.^67^

80/50 K Mössbauer spectroscopy of as-prepared FeNC materials revealed three doublets, D1, D2, and D3, characteristic for this class of materials. Air exposure of the FeNC materials leads to an increase of D1 and a decrease of D2 and D3, indicating ligand change by oxygen binding and a partial oxidation of ferrous iron, respectively. EPR showed an increase in the dominating superparamagnetic EPR signal and an increase in rhombic HS Fe­(III) sites for FeNC (+air).

These findings indicated that despite post-preparation treatments (e.g., acid leaching) to remove iron oxides, superparamagnetic iron oxide was present in the as-prepared materials, and its content further increased under air. Strongly and weakly coupled octahedral HS Fe­(III) in iron oxide structures were assigned major contributions to D1. These structures are either formed already during synthesis or under air from reduced ferrous iron sites in the carbon matrix. Regarding FeN_4_ environments, D1 has in the past been assigned to HS Fe­(III), or LS Fe­(II).^21,22^ Despite that the Mössbauer parameters of D1 are in good agreement with HS Fe­(III), the absence of the characteristic EPR signal questions the assignment to ferric FeN_4_ macrocycles with square-pyramidal or octahedral coordination. Upon lowering the temperature below 5 K in N_2_, D1 largely splits up to sextets (∼2/3 of D1 at RT), again indicating that a large contribution to D1 are exchange-coupled iron oxide sites. The remaining D1 contribution that does not split up even below 2 K in a N_2_ environment could possibly contribute from isolated or weakly coupled LS Fe­(II) or being associated with small iron oxide clusters that even at 1.8 K do not reach full magnetic ordering.

When the potential of FeNC electrodes exposed to air in N_2_-saturated electrolyte was reduced to 0.2 V, D1 in the RT SEC-Mössbauer decreased to less than 50% of its original integrated area at 0.9 V. 23% of the loss in D1 was attributed to leaching. Importantly, the decrease in D1 was accompanied by a comparable increase in D3, and this change in D1 and D3 was reversible. As soon as the potential was switched back to 0.9 V, D1 increased and D3 decreased. This indicates a reversible redox cycle between Fe­(III) and Fe­(II) states in the iron oxide moieties bound in the FeNC catalyst. SEC-EPR, which showed a reversible decrease and increase in the rhombic Fe­(III) EPR signal under the same potential changes, supports this assignment.

To verify this assignment, in Figure 8, we compare Mössbauer parameters obtained in this work with values reported for iron oxides in glasses and in FeN_4_ macrocycles. For iron oxide in glasses, only high-spin states are reported, limiting the possible areas to two ranges representing a tetrahedral or octahedral type of coordination. For molecular FeN_4_ moieties depending on the kind and number of axial ligands, all spin states are possible. For the sake of simplicity, only Mössbauer values of Fe­(II) and Fe­(III) are displayed, taken from ref 11 with uncertainties of 0.05 and 0.1 mm s^–1^ for CS and Δ*E*_Q_, respectively. An overview of Mössbauer parameters obtained in previous studies are summarized in Figure S19 in addition to iron oxides present in glasses. In Figure S20, experimental SEC-Mössbauer spectra from the literature are shown that were simulated with our fit model.

**Figure 8:**
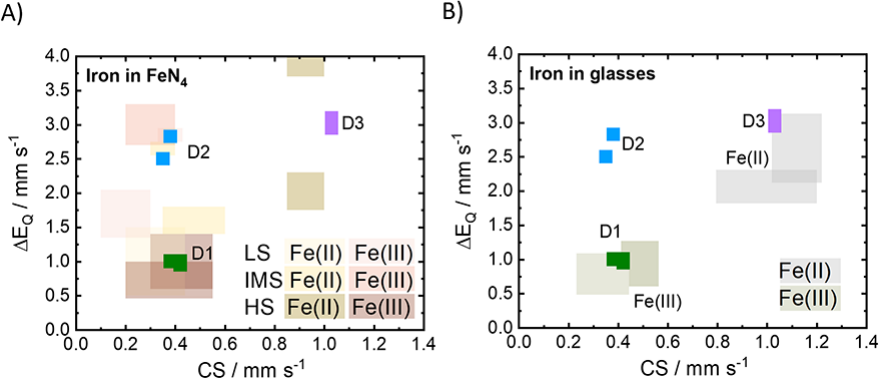
CS and Δ*E*_Q_ for D1, D2, and D3 in comparison to (A) values obtained for Fe­(II) and Fe­(III) in FeN_4_ macrocycles^11^ at RT and (B) iron oxide structures in glasses.^38,61^ See SI Figures S19 and S20 for comparison to other works on *in situ*/*operando* SEC-Mössbauer spectroscopy.

In a previous study, Saveleva et al.^20^ using spectroelectrochemical X-ray emission spectroscopy on the K-β′ mainline observed a reversible decrease of the average spin state under applied potential of 0.2 V as compared to 0.9 V. This is in line with the significant increase of HS Fe­(III) observed at 0.9 V herein. In our study, after a potentiostatic hold at 0.2 V, only a fraction of about 14% of the initial D1 did not recover after reapplying 0.9 V, corresponding to a comparable reduction of the HS Fe­(III) EPR signal. D2 and D3 have in the past been assigned by their CS and Δ*E*_Q_ values to IMS Fe­(II) and HS Fe­(II) FeN_4_, respectively.^11^

The Mössbauer parameters of D2 are similar to those of IMS Fe­(II) in FeN_4_ such as β-Fe phthalocyanine.^68,69^ The assignment of D2 to a FeN_4_ site is in accordance with previous findings;^17,48^ in some of them, it was considered an active site in ORR,^17,18,70^ while others classified it as inactive.^71^

In the present work, in 0.2 V SEC-Mössbauer spectra, an absolute increase in D2 of 5% relative to the absolute integrated area of the initial 0.9 V spectrum was observed (Figure 7D). This may indicate an involvement of D2 in the redox process; however, since the obtained changes are in the same range as the error margins of our analysis, no clear assignment is possible. EPR gave no indication of ferric HS FeN_4_ macrocycle-type coordination. Based on these findings, the hypothesis that the reversible potential-dependent changes of D2 involved a ferrous FeN_4_ can only be maintained under the assumption of an EPR-silent FeN_4_ contribution to D1.

A possible scenario, though not accessible with the methods applied here, would be that although the majority of D1 consists of HS Fe­(III) (likely in iron oxide environments), a minor D1 contribution (this could be the 14% unrecovered D1, see Figure 7C) is from LS Fe­(II)­N_4_ that experiences a coordination-induced spin-state change to IMS Fe­(II) upon lowering the potential to 0.2 V. Under this assumption, the oxidation state would remain, and the iron would transition from an EPR silent state to another. Ligand change-induced spin state changes are known for, e.g., Fe macrocycles,^70,72^ and involve a change of the axial ligand(s). The possibility of spin-state change in the FeN_4_ moieties of FeNC materials was postulated by Li et al.^18^ with data based on combined SEC-Mössbauer spectroscopy and quantum chemical calculations. In their work, similar but not identical MS parameters were found. Mainly, the chemical shifts of D2 and D3 were lower compared to our values (Figure S19A).^72^

The Mössbauer parameters of D3 are similar to 6-fold coordinated Fe­(II) in iron oxides, e.g., AC or NPs,^38^ while there is no match of molecular FeN_4_ moieties to D3. Therefore, the observed reversible switch between D1 and D3 may be associated with a redox transition between Fe­(III) and Fe­(II) in iron oxide particles, as D3 already contributed to the Mössbauer spectra of the as-prepared FeNC material (N_2_), but no NPs were identified in HRTEM images prior to air exposure. If D3 originates from Fe­(II) in oxide structures, it is more likely to be located in AC-type structures.

Xu et al.^19^ investigated FeNC produced by pyrolysis and subsequent acid leaching using SEC-Mössbauer to study demetalation of FeN_4_ sites. The Mössbauer spectra were fitted with doublets that were in parts similar to those in our study (Figure S19B) and assigned to different pyrrolic and pyridinic FeN_4_ environments. However, since no EPR and low-temperature Mössbauer spectra were obtained, it is difficult to clearly distinguish between the assigned FeN_4_ sites and the possible existence of iron oxide structures identified herein.

In our own previous SEC-Mössbauer spectroscopy^16^ work on another FeNC catalyst, D1 was assigned to bare sited IMS Fe­(II)­N_4_C_12_ or hydroxide-bound HS Fe­(III)­N_4_C_12_ and D3 to HS (*S* = 2) Fe­(II)­N_4_C_12_ with OOH^–^ or OH^–^ ligands. Both sites were regarded as key contributors to ORR activity among various Fe sites with the identification of an additional intermediate D4 as dioxygen-bound Fe­(II)­N_4_C_12_ or hydroperoxide-bound Fe­(III)­N_4_C_12_ formed during catalysis.

The data showed that the deoxygenated environments associated with D2 and D3 correlated with another site, only present under the *operando* condition. This D4 site clearly lays out the ranges of iron oxide environments (Figure S19C) and is correlated in intensity with the ORR activity of that catalyst. Based on this, and the reversibility of switching, it is unlikely that the electrochemically active iron sites identified in Ni et al.^16^ originate from iron oxide. Nonetheless, it is evident from LT MS that the catalyst contained also significant fractions of iron oxide impurities, which however did not change during applied potential.

In a related SEC-Mössbauer study on a different FeNC catalyst (Fe_0.5_), Li et al.^17^ reported potential-dependent trends in the Mössbauer spectra, which are qualitatively in accordance with our observations (Figure S20D). However, a different assignment was made based on the experimental data. The observed D1 was assigned to HS O_2_–Fe­(III)­N_4_C_12_ located in the amorphous carbon network and D2 to LS (*S* = 0) or IMS (*S* = 1) Fe­(II)­N_4_C_10_ in graphitic carbon. D1H (same parameter as our D1 under *ex situ* condition) was reversibly converted to D1L (HS, Fe­(II)­N_4_C_12_) with CS = 0.79 ± 0.11 mm s^–1^, Δ*E*_Q_ = 2.0 ± 0.01 mm s^–1^ by decreasing the potential to 0.2 or 0.4 V under argon. D3 (CS = 1.17 ± 0.06 mm s^–1^, Δ*E*_Q_ = 2.57 ± 0.12 mms^–1^) was also identified under low potential and was found to be unaffected by potential changes. D3 was assigned to HS Fe­(II) that was oxidized to Fe_2_O_3_ after air exposure.

During 50 h of operation at 0.5 V in H_2_/O_2_ PEMFC, D1 was assigned as the active site for ORR but found to quickly degrade into iron oxides. D2 was found unchanged at potential holds and was assigned as a persistent catalytic site for the ORR.^17^ Taking into account the uncertainty in the assignment of the Mössbauer sites due to the low resolution of the spectra generally encountered in pyrolyzed FeNCs and the large contribution of iron oxides to D1, which became visible only in Mössbauer spectra below 5 K in the present study, we regard it plausible that a larger amount of iron oxides was present in the Fe_0.5_ materials studied by Li et al.,^17^ as the same catalyst in another work of the authors also showed the characteristic EPR signature of iron oxide.^20^ There are two main differences between our assignments and ref 17, though we observed similar sites and trends. The first is the assignment of D1 to O_2_–Fe­(III)­N_4_, whereas we assign this to mainly HS Fe­(III) in iron oxides. Second are the assignments of D1L and D3 to HS Fe­(II)­N_4_C_12_ and HS Fe­(II), respectively. However, when compared to iron oxides in glass, we find that both D1L and D3 have Mössbauer parameters in the range of Fe­(II) in iron oxides.^61^ Therefore, the observed potential-dependent changes of the Mössbauer spectra may have also originated from reduction and oxidation of iron oxide structures.

Finally, we fitted our model to the spectra obtained in the aforementioned works,^16–19^ to see if their spectra above and below the onset potential (above: 0.9 V or OCP; below: 0.2 or 0.3 V, all in deaerated condition) can be reproduced assuming the same set of Mössbauer parameters. The respective fits are listed in Figure S20. Overall, there is a relatively good match, indicating the possibility of similar iron oxide species. However, the residuals of experimental vs calculated spectra indicate that an additional doublet (similar to a Fe­(II) HS moiety) would be required to enable a full match. This suggests that reversibly switching, redox-active iron oxide structures may also be present in other FeNC catalysts, but their proportion to FeN_4_ moieties could vary depending on the preparation. The extent to which such iron oxide moieties also contribute to ORR activity or whether they influence the catalytic activity of other catalytic Fe sites requires further experimental work.^71^ Figure 9 gives a schematic overview of Fe structures found in our study.

**Figure 9:**
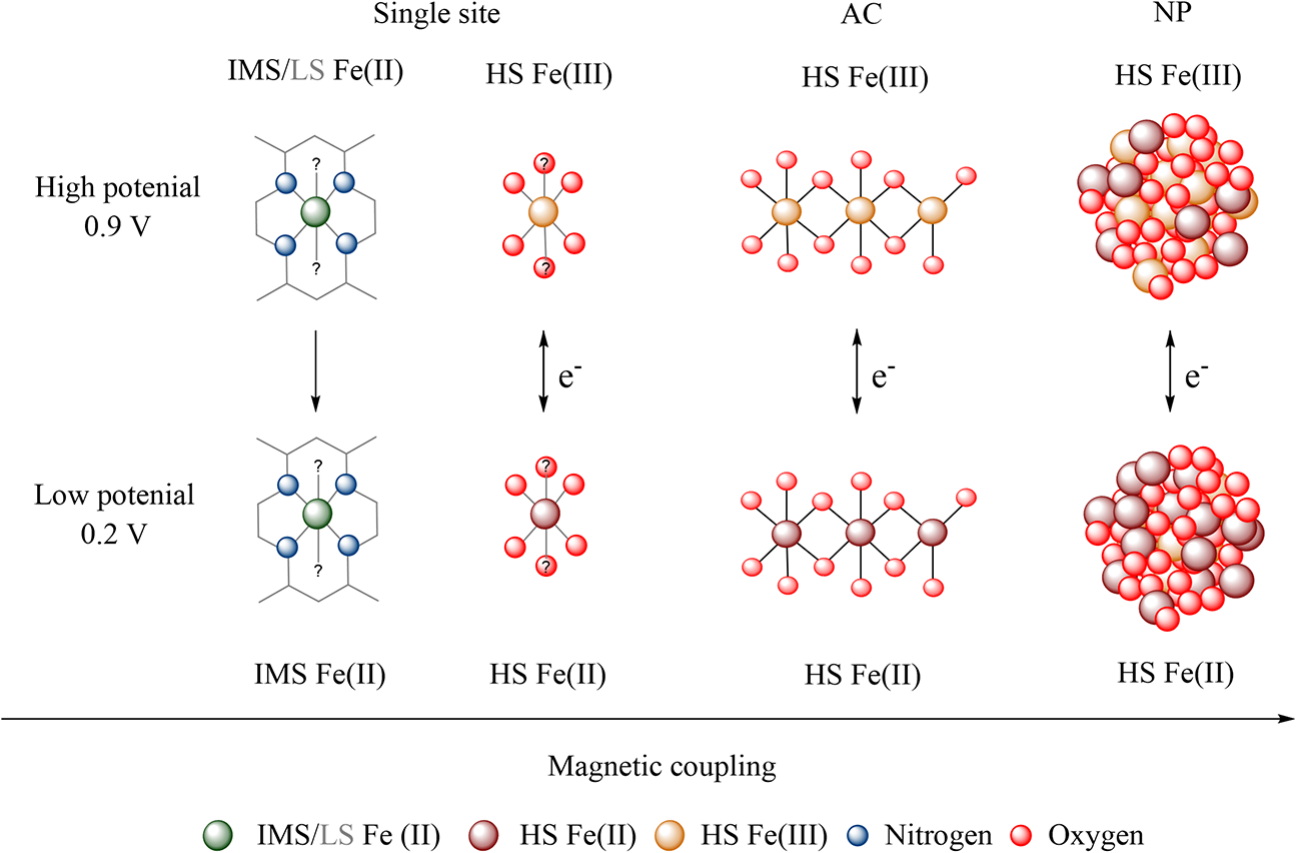
Summary of Fe structures identified in the FeNC catalysts studied in this work, ordered along their increasing magnetic couplings from single sites to AC and NPs type iron oxide structures. In FeNC material, the majority of the Fe is bound as HS Fe­(III) and HS Fe­(II) in an oxidic coordination environment and as IMS Fe­(II) in FeN_4_ sites. Upon lowering the potential oxidic HS, Fe­(III) is partly reduced to HS Fe­(II). In addition, upon lowering the potential, LS Fe­(II)­N_4_ sites may undergo ligand change-induced spin change to IMS Fe­(II).

## 5 Conclusions

Microscopic and spectroscopic characterization of a state-of-the-art FeNC catalyst revealed a distribution of distinct Fe environments ranging from isolated Fe sites to a few atoms containing atomic clusters and larger iron oxide nanoparticles. The isolated and clustered sites are present in prepared samples in an inert atmosphere. Upon exposure to air, isolated sites are converted to AC and NPs alongside a significant oxidation from LS Fe­(II) to HS Fe­(III). These observations indicate that the post-treatments to remove iron oxides were insufficient as they are reformed as soon as the catalysts are exposed to oxygen. A similar trend was even observed during long-term storage in liquid nitrogen, indicating continuous structural changes.

Complementary, EPR and Mössbauer spectroscopy revealed that a variety of iron oxide structures are the main cause of the characteristic Mössbauer doublets D1 and D3 of this FeNC. SEC-Mössbauer spectroscopy showed that D1 and D3 exhibit opposite changes under an applied potential, which indicates reversible redox-active iron oxide structures in the FeNC materials investigated. Low-temperature EPR additionally detected an HS Fe­(III) site in rhombic distorted octahedral coordination, which could be reversibly oxidized and reduced in the SEC-EPR experiments. This signal is attributed to isolated or weakly coupled HS Fe­(III), presumably in iron oxide structures. The remaining Mössbauer doublet, D2, was attributed to EPR-silent IMS Fe­(II) in an FeN_4_ site. This site showed only minor changes upon contact with air and the electrochemical potential. Its involvement in the observed redox behavior could not be clearly assigned.

Despite the performance of Mössbauer in identifying iron sites, we demonstrate that a combination of EPR and Mössbauer experiments, both below liquid He temperatures and at room temperature as well as under spectroelectrochemical conditions, are crucial to elevate ambiguity in the discrimination of isolated sites and iron oxide structures. Our findings suggest that iron oxide structures in FeNC may not only be degradation products but react reversibly upon applied potential. This points toward a previously unconsidered possible contribution to the ORR over FeNCs and may lead to new optimization strategies targeting improved performance and in particular stability in FeNCs.

## Supplementary Material



## Data Availability

Raw data of Mössbauer and EPR spectroscopy, the EPR spectral fitting model, script and stick spectra for species with large distribution in zero-field parameters E and D, and the TEM images of FeNC material can be found in https://doi.org/10.17617/3.WIW7CI.

## Notes

Developed spectrolectrochemical EPR setup, additional data on the electrochemical characterization of the FeNC electrodes, further microscopy images, EPR and Mossbauer spectra of FeNC materials and electrodes, detailed description of the simulation routines used to model EPR and Mössbauer, and visualization and simulation of FeNC literature data (PDF)

## Abbreviations

PGM: platinum group metal
MNC: metal nitrogen carbon catalysts
DOE: Department of Energy
XAS: X-ray absorption spectroscopy
EPR: electron paramagnetic resonance spectroscopy
TEM: transmission electron microscopy
RT: room temperature
CS: chemical shift
ΔE_Q_: quadrupole splitting
SEC: spectroelectrochemical
SEC-EPR: spectroelectrochemical EPR
AL: acid leached
RRDE: rotating ring disk electrode
WE: working electrode
CE: counter electrode
RE: reference electrode
POM: polyoxymethylen
ADF: annular dark field
STEM: scanning transmission electron microscopy
EDX: energy dispersive X-ray
HRSTEM; AC: high-resolution scanning transmission electron microscopy; atomic clusters
NPs: nanoparticles
