# Supplementary material for: Reversibly Redox-Active Iron Oxide Structures in FeNC Catalysts Identified by Microscopy and Spectroelectrochemical EPR and Mössbauer Methods
Source: J Am Chem Soc. 2026 Jan 10;148(4):3995–4007. doi: 10.1021/jacs.5c12396 (PMC12879734; doi:10.1021/jacs.5c12396)
Supplement: Supplementary file 1 [file ja5c12396_si_001.pdf]

Reversibly Redox Active Iron Oxide structures in FeNC Catalysts Identified by Microscopy and Spectroelectrochemical EPR and Mössbauer Methods

Kaltum Abdiaziz[1#], Lingmei Ni[2#], Derya Demirbas[3], Hendrik Haak[2], Edward Reijerse[1], Pascal Theis[2], Wulyu Jiang[4], Sonia Chabbra[1], Thomas Lunkenbein[4], Ulrike I. Kramm[2,*], Alexander Schnegg[1,*]

[1] *Max Planck Institut für Chemische Energiekonversion, Mülheim an der Ruhr 45470, Germany*

[2]*Catalysts and Electrocatalysts Group, Department of Chemistry, Technical University, Darmstadt*

64287, Germany

[3]*Max–Planck–Institut für Kohlenforschung, Mülheim an der Ruhr 45470, Germany*

[4]*Fritz–Haber–Institut der Max–Planck–Gesellschaft, Berlin 14195, Germany*

Contents

1. Materials and Methods 3
   - 1.1 Sample preparation 3
   - 1.2 Microscopy 4
   - 1.3 Inductively coupled plasma emission spectroscopy (ICP-OES) 4
   - 1.4 Electrochemistry 4
   - 1.5 EPR spectroscopy 5
   - 1.6 Mössbauer spectroscopy 6
2. Electrochemical conditions for SEC-EPR and SEC-MS 7
3. EPR Spectroscopy 12
4. Mössbauer Spectroscopy 16
5. Microscopy 20
6. SEC-Mössbauer Spectroscopy 23
7. Comparison of Mössbauer parameters (CS and ΔE_Q) to literature 28

1. Materials and Methods

1.1 Sample preparation

Materials: All reagents were commercially available and were used without further purification. The isotopically enriched ^{57}Fe metal powder (> 95 % Fe-57) from CHEMGAS, 5 wt % Nafion solution NS-5 from Quintech GmbH (PFSA 5 wt %, D521), sulfanilic acid from Carl Roth (> 98 %). All other chemicals were obtained from Sigma Aldrich. Ultrapure water (18.2 MΩ cm) was used to ensure no cross-contamination. For both, SEC-Mössbauer and SEC-EPR the identical isotopically enriched ^{57}FeNC catalyst was used. The preparation is described in the following steps;

1.1.1 $^{57}FeCl_3$ synthesis

The synthesis of anhydrous $^{57}FeCl_3$ followed the protocol outlined by Heppe *et al.*[1] Concentrated hydrochloric acid and ultrapure water were degassed (N_2) for 15 minutes before use. Subsequently, a defined quantity of ^{57}Fe (7.094 g, 0.125 mol) was suspended in ultrapure water (100 mL) introduced in a three-neck flask. With continuous stirring in nitrogen atmosphere, concentrated (37 wt%) hydrochloric acid (101 mL, 1.214 mol) was gradually added using a dropping funnel over a span of 30 minutes. The mixture was then heated to 60 °C and stirred until complete iron dissolution, as indicated by the absence of visible bubbles. Once cooled to RT, 122 ml of a 35 wt% hydrogen peroxide solution was introduced through a dropping funnel. The solution was then heated to 60 °C for one hour, then dried at 120 °C. For the production of anhydrous ^{57}Fe(III) chloride, the obtained precipitate was combined with thionyl chloride ($SOCl_2$, 89 ml, 1.230 mol) whilst stirring. The solution was stirred for one hour and then dried at 100 °C. The product obtained (dark green solid) was stored under nitrogen atmosphere. The achieved yield is 96.6 % (19.904 g).

1.1.2 Precursor synthesis

In the synthesis of the precursor compound polypyrrole, ultrapure water (18.2 MΩ cm, 500 ml) was cooled to 3° C, degassed with N_2 for eight minutes and split into a round-bottomed flask (400 ml) and a beaker (100 ml). Sulfanilic acid (0.436 g, 2.5 mmol) and pyrrole (3.6 ml, 52 mmol) were added to the round bottom flask and dissolved with the aid of ultrasonic bath. Parallel to this step, ^{57}Fe(III) chloride (8.0 g, 49.0 mmol) were dissolved in the water in the beaker. The dissolved ^{57}Fe(III) chloride was added into the round bottom flask mixture whilst swirling. The flask was then stored at -10 °C for 17 hours. Then, the round bottom flask was thawed at room temperature for seven hours. The resulting crude polypyrrole (dark brown to black) was obtained through filtration. Post-filtration, any residual water was eliminated by drying at 80 °C for 18 hours. The product was homogenized by grinding in a stainless-steel mortar until homogeneity. The final product (black solid) yielded 35% based on the initial amount of pyrrole used. It was noted, that beside polypyrrole the product intentionally contained iron chloride and sulfanilic acid residuals.

1.1.3 Catalyst preparation

For the preparation of the FeNC catalyst, 1.200 g of the self-made polypyrrole precursor and 1.890 g ^{57}Fe(III) chloride (stored in the absence of air) were combined in a 35 mL zirconia grinding beaker containing four zirconia balls (10 mm diameter). The mixture was homogenized at 30 Hz for 15 minutes in a ball mill and then dried in a Petri dish for 20 minutes at 80 °C in a drying oven. The mixture was then filled into inert alumina boats and placed in a quartz glass tube in the furnace. The first heat treatment (1st HT) was carried out entirely in an inert (N_2) atmosphere with a temperature ramp of 300 °C h^{-1} up to 800 °C, holding time at 800 °C for one hour and natural cooling of the sample. After the cooling step, the catalyst was acid leached (1st AL) by placing the inert boats with the pyrolysis product in a degassed (N_2) 2 M hydrochloric acid (300 mL) and 2-propanol (75 mL). The addition of 2-propanol helps to get a good dispersion.[2] To accelerate the acid leaching, the round bottom flask was treated for 150 minutes at 50 °C in an ultrasonic bath, and was then allowed to rest for an additional 14 hours at room temperature. For the period of acid treatment, the inert atmosphere of the flask was maintained by continuously saturated with nitrogen. To isolate and process the product obtained, the dispersed particles were filtered through Durapore membrane filters (Merck Millipore, Burlington, USA, type 0.45 µm HV), washed ten times with 50 mL of water each, dried in a drying oven at 80 °C for ten hours, and homogenized in an agate mortar for three minutes. The second temperature treatment (2nd HT) was performed with a temperature ramp of 800 °C h^{-1} from room temperature to 800 °C, a holding time at 800 °C for 15 minutes and then natural cooling of the sample. The second heat treatment was carried out in inert (N_2) atmosphere from RT to 600 °C and in the cooling process. Between 600 °C and 800 °C, forming gas (10% H_2/ 90% N_2) was used instead of nitrogen. Following the second temperature treatment, a second acid leaching was

performed in a solution of 2 M hydrochloric acid (200 mL) and 2-propanol (50 mL). From previous work it is known, that this procedure enables a significant improvement of the purity of the catalyst.[3] The catalyst was filtered, washed and dried before a third heat treatment (3rd HT), with a temperature ramp from 800 °C h^{-1} to 800 °C, a holding time at 800 °C for 15 minutes and then natural cooling of the sample. All steps of this temperature treatment took place exclusively in an inert atmosphere. Following this temperature treatment, the product obtained (black solid) yielded 53.6% (1.28 g) based on the initial amount of polypyrrole precursor used.

1.2 Microscopy

TEM, STEM, and EDX analyses were conducted using a FEI/Thermo Fisher Scientific (TFS) Talos F200X operated at 200 kV, equipped with a FEI/TFS Ceta 16M CMOS camera. Elemental analysis and quantification were performed using the integrated four-quadrant silicon drift detectors (SDDs). The data was processed via Velox software.

For atomic-resolution STEM imaging, a JEOL ARM 200F operated at 200 kV was employed, featuring CEOS CESCOR and CETCOR double aberration correctors. To enhance the signal-to-noise ratio, multiple frames were acquired over a two second interval and summed with online drift correction applied using GMS3 software (Gatan). The brightness or contrast variations in HR-STEM images arise from atomic densities and atomic number (Z-contrast) as such iron atoms, with their specific electron density and Z-number, can be distinguished from lighter elements (carbon, nitrogen and oxygen in this case) due to their higher scattering intensity. The gray-white contrast we observed could also be due to noise, inelastic scattering, or variations in sample thickness as such we look for clear contrast difference which can be as iron atoms. The atomic-resolution STEM images as in Figure 3, were acquired through sampling over different regions of the FeNC catalysts.

1.3 Inductively coupled plasma emission spectroscopy (ICP-OES)

Inductively coupled plasma emission spectroscopy (ICP-OES) was carried out with a Spectro Genesis FES 27 (Ametek Inc.). For the measurement, 10 mg of the sample was dissolved in a mixture of 66 mL of concentrated sulfuric acid (98 %, Kraft GmbH) and 33 mL hydrogen peroxide (35 %, Grüssing GmbH). After an initial strong reaction, the resulting mixture was heated under reflux for 12 h until a clear yellow solution was obtained. The resulting solution was analyzed with the ICP-OES system for iron content utilizing the λ = 259.9 nm Fe spectral line. Calibration was done with a five-point calibration in the range of 0 µg L^{-1} to 10 mg L^{-1} iron content.

1.4 Electrochemistry

1.4.1 Catalyst ink preparation for RRDE measurement

5 mg of FeNC material was added to 142 µL ultrapure H_2O (18.2 MΩ cm), 25 µL Nafion solution and 83 µL isopropanol. Before drop casting the ink onto the electrode, the ink solution was first kept in an ultrasonic bath with ice for 45 min and then treated with a Vortexer for 1 min followed by another 15 min ultrasonic bath treatment.

1.4.2 Standard RRDE-experiments protocol

All standard electrochemical experiments were performed in a three-electrode setup with working electrode, Ag/AgCl reference electrode and a glass carbon rod counter electrode. The working electrode (rotating ring disk electrode (RRDE)) is combined of a glassy carbon disc equipped with a Pt ring. The disk serves as support for the deposition of the catalyst ink (working electrode) and a ring electrode that serves as a 2nd working electrode for H_2O_2 detection. ORR tests were conducted with a Parstat3000A (AMETEK) potentiostat in N_2 and O_2 saturated 0.1 M H_2SO_4 electrolyte.

For the test protocol, cyclic voltammetry curves were first conducted in nitrogen saturated electrolyte at a scan rate of 300 mV s^{-1} from 1.1 to 0 V for 20 cycles to clean the surface. Simultaneously, the ring electrode was activated by the same cycling procedure. Then each time one cycle with 100 mV s^{-1}, 50 mV s^{-1}, and 10 mV s^{-1} was carried out in the same potential range. Afterwards the electrolyte was saturated with oxygen and the ORR activity was measured by performing cyclic voltammetry in the same potential window with a sweep rate of 10 mV s^{-1} and with different rotation speeds of 0, 200, 400, 900, 1500, and 2500 rounds per min (rpm). The potential for the ring electrode was fixed to 1.2 V to oxidize any H_2O_2 generated at the disk. The hydrogen peroxide yield was calculated from the disk current I_D, the ring current I_R and the collection efficiency (N = 0.38, as provided by the manufacturer):

$$\mathrm{H_2O_2\ in\ \%} = 100 \cdot \frac{2I_\mathrm{R}}{|I_\mathrm{D}| \cdot N + I_\mathrm{R}}$$

1.5 EPR spectroscopy

1.5.1 X-band and Q-band CW EPR spectroscopy

Low temperature (< 100 K) X-band continuous wave (CW) EPR measurements were performed using a Bruker E500 ELEXSYS spectrometer equipped with a Bruker dual-mode resonator (ER 4116DM, Bruker, Germany), Oxford Instruments helium flow cryostat (ESR 900) and Mercury iTC temperature controller (Oxford Instruments, UK). EPR measurements were conducted at 10 - 100 K with 0.2 mW microwave (mw) power, 100 kHz modulation frequency and 7 G modulation amplitude. X-band EPR between 100 - 300 K was measured in a Bruker MS 5000 spectrometer equipped with variable temperature unit with 0.2 mW mw power, 100 kHz modulation frequency and 7 G modulation amplitude. Q-band (~34 GHz) CW EPR spectra were conducted using a Bruker Elexsys E580 EPR spectrometer, equipped with a laboratory-built Q-band extension. The following instrument parameters were used for Q band EPR; 0.02 mW power, 100 kHz LockIn modulation frequency and 7 G modulation amplitude. Cryogenic temperatures at Q-band were achieved with an Oxford CF935 cryostat. EPR spectra were simulated with scripts based on the Matlab^{TM} toolbox EasySpin,[4] as described in the SI. FeNC material (18-20 mg) were filled into 3.8 mm EPR quartz tubes in the glovebox and stored under liquid nitrogen (N_2 and +N_2LTS) and then exposed to air at ambient conditions for 10 minutes (+air) before freezing the sample again in the cryostat of the EPR spectrometer.

1.5.2 SEC-EPR

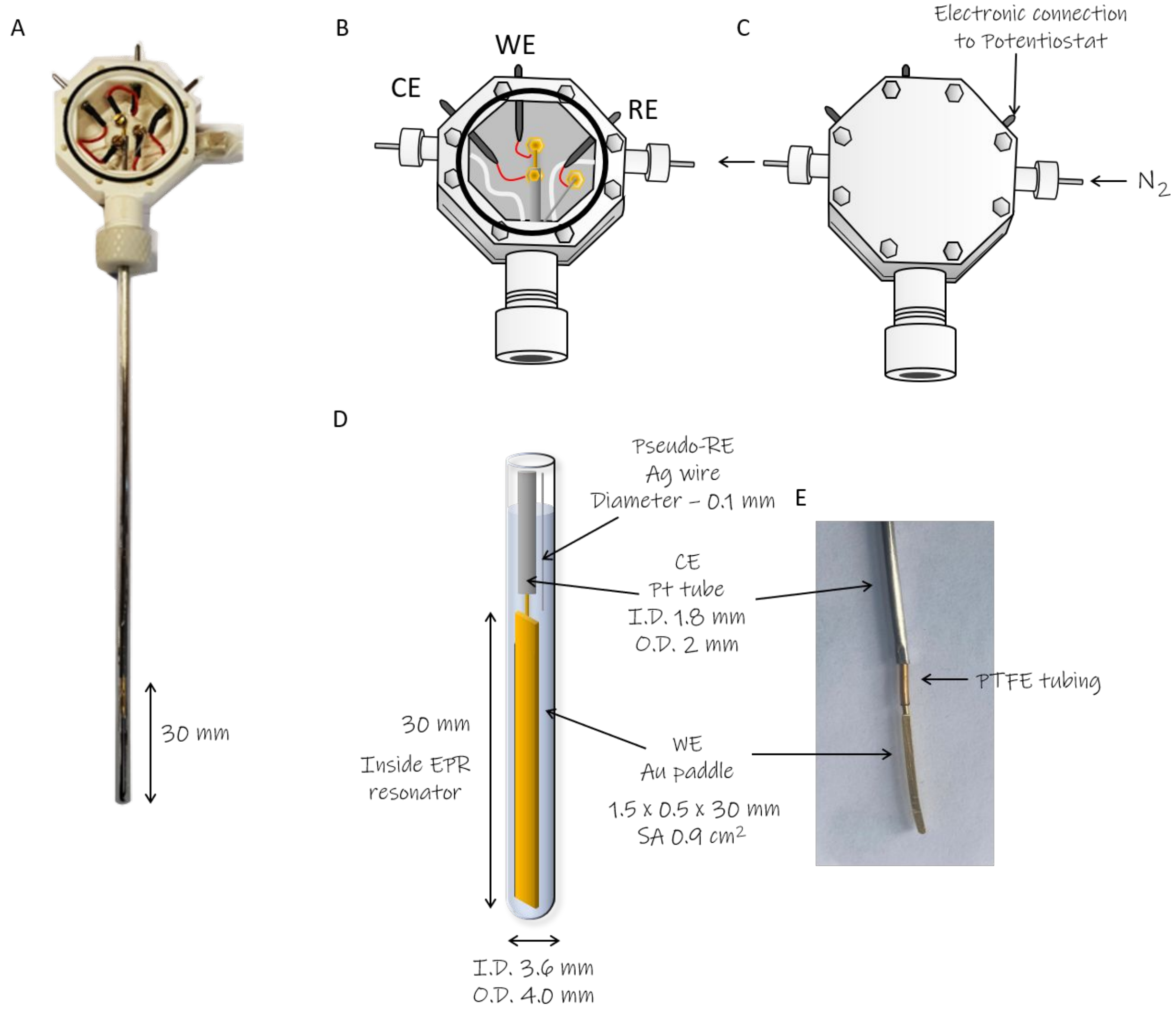

Figure S1. (A) Photograph and (B) schematic diagram of 3D printed probe head without and (C) with lid. (D) Schematic diagram of 3-electrode configuration SEC-EPR tube. (E) Photograph of the working and counter electrodes.

Figure S1 shows photographs and schemes of the SEC-EPR setup. Though a Bruker E500 ELEXSYS spectrometer was used for *in* SEC-EPR in this work, the setup is adaptable for any cryogenic EPR spectrometer which accommodates 4 mm tubes or larger. The design of the EPR cell was optimised for electrochemical experiments on paramagnetic specimen in materials deposited on the widened working electrode (WE). For this reason, the electrode was placed inside the EPR resonator in the magnetic field maximum of the microwave field. Experiments with this configuration showed that it was well suited for EPR experiments on electrochemically generated paramagnetic centres (radicals and metal ions) in frozen solution[5] or deposited on the WE.[1] The cell followed a three-electrode configuration in an EPR quartz tube (I.D. 3.6 mm O.D. 4.0 mm, Length 135 mm). The SEC-EPR cell consists of two sections. The upper section is a 3D printed probe head with a lid and electrical connections for the electrodes (Figure S1B and C). The electrodes and lid were secured using bolts and made air tight using an O-ring between lid and probe head. A PTFE flexible tube (I.D. 1. mm O.D. 0.8 mm) was used for gas exchange and placed through a gas inlet and outlet in the probe head. Pins were connected to the probe head for ease of connection to the potentiostat. The bottom section consists of the EPR quartz tube and the three electrodes (Figure S1D). Gold wire (diameter 1 mm, lengths 250 mm) with the bottom 30 mm (height) flattened to a length of 1.5 mm width and a width of 0.5 mm length was used as the WE. The FeNC material was deposited on the front-face and the back face of the WE (A = 0.9 cm^2). This bottom 30 mm is placed inside the EPR resonator to enable EPR measurements at cryogenic temperatures. For the frozen solution configurations tested the Q-factor of the resonator was not affected. The WE was placed inside the platinum tube for stability (Figure S1E). To prevent short circuit and limit electrical connection between the working and counter electrode, the upper wire section was insulated using PTFE heat shrink tubing. Insulated Ag wire (diameter 0.1 mm, lengths 250 mm) with the bottom 5 mm uncoated was used as the reference electrode and the counter electrode was a platinum tube (I.D. 1.8 mm O.D. 2.0 mm length 200 mm). Ag wire is used instead of a traditional reference electrode due to space constraints in the EPR tube. Prior to any SEC-EPR measurements, the offset was measured for the Ag wire and calibrated against a standard Ag/AgCl (0.3 M KCl) reference electrode, offset was found to be 0.224 V vs RHE (reversible hydrogen electrode). Gold electrode was chosen as the WE for its malleability, inertness and EPR silent character. Electrodes were cleaned with ultrasonication in isopropanol and water and calcinated using a hot flame before use. The three electrodes were positioned in the EPR tube as shown in Figure S1D.

1.6 Mössbauer spectroscopy

1.6.1 ^{57}Fe Mössbauer (ex situ)

^{57}Fe Mössbauer spectra recorded at and below 80 K were carried out on a different spectrometer than at 298 K with low-temperature spectra acquired first, followed by the 298 K spectrum. For 298 K; the spectra were recorded on a spectrometer equipped with an Oxford Instrument; Variox cryostat. Measurements below 80 K at zero field were carried out with a cryogen-free magnet system with an integrated variable temperature insert (VTI) from Cryogentic Ltd. The velocity axis was calibrated against high-purity α-iron foil with 12µm thickness at 298 K. The ^{57}Co source in Rh matrix (1.85 GBq) was placed and positioned inside the gap of the magnet system (re-entrant bore) at room temperature (298 K) and zero field position. An uncertainty of ± 0.02 mm s^{-1} is to be expected due to fitting of the calibration data. For the measurements, ^{57}FeNC material (18-20 mg) was filled into a 1 cm^2 Delrin (Polyoxymethylen (POM)) sample holder and mounted in front of the detector. This accounts for 5 mg cm^{-2} sample, which is close to the optimum in terms of thin layer approximation. The minimum experimental line width measured as the full width at half-maximum was 0.24 mm s^{-1}. The Mössbauer spectra were simulated and fitted using MF2 Zero-field Mössbauer spectra written by Eckhard Bill[6] and Recoil software.[7] Both software and fitting models employed are discussed further in Section 5 of the SI.

2. Electrochemical conditions for SEC-EPR and SEC-MS

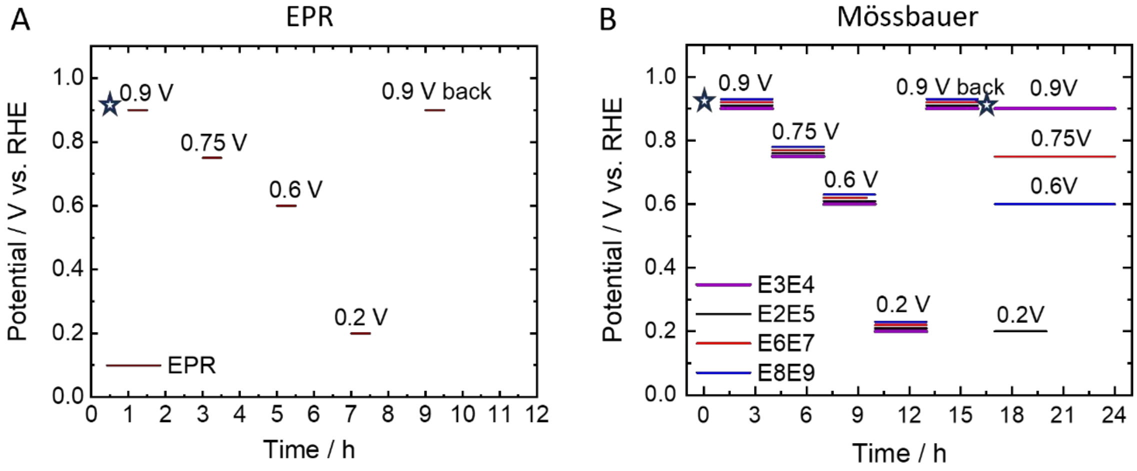

Figure S2. Measurement protocols for (A) quasi *in situ* EPR and (B) *in situ* Mössbauer of FeNC electrodes (+air) in N_2 saturated 0.1 M H_2SO_4. Cyclic voltammetry was carried out in the SEC-EPR setup, prior to *in situ* measurements (A - star). SEC-EPR measurements were conducted sequentially at potentials of 0.9 V, 0.75 V, 0.6 V, 0.2 V, and then returned to 0.9 V at room temperature. Each potential was held for 20 mins, flash frozen in liquid N_2 and transferred to EPR spectrometer for measurements. For SEC-Mössbauer, two electrodes were each loaded with 20 mg of catalyst and combined to function as the WE due to the low iron content of only 0.2 wt %. For instance, in the E3E4 electrode set, measurements were conducted at potentials of 0.9 V, 0.75 V, 0.6 V, 0.2 V, and then returned to 0.9 V. Each potential was maintained for three hours, with the 0.9 V potential selected for extended overnight measurement (7 hours). For the other sets, different potential hold at night were selected (7 hours for 0.75 V and 0.6 V and 3 hours for 0.2 V). For E2E5 electrode, due to an operational oversight, the measurement was conducted for only three hours instead of the intended overnight. Cyclic voltammetry measurement (B - star) were conducted on each electrode set before the potential hold at 0.9 V and after the potential hold at 0.9 V back. The *in situ* Mössbauer spectrum at a specific potential represents the cumulative sum of all subspectra recorded at that potential.

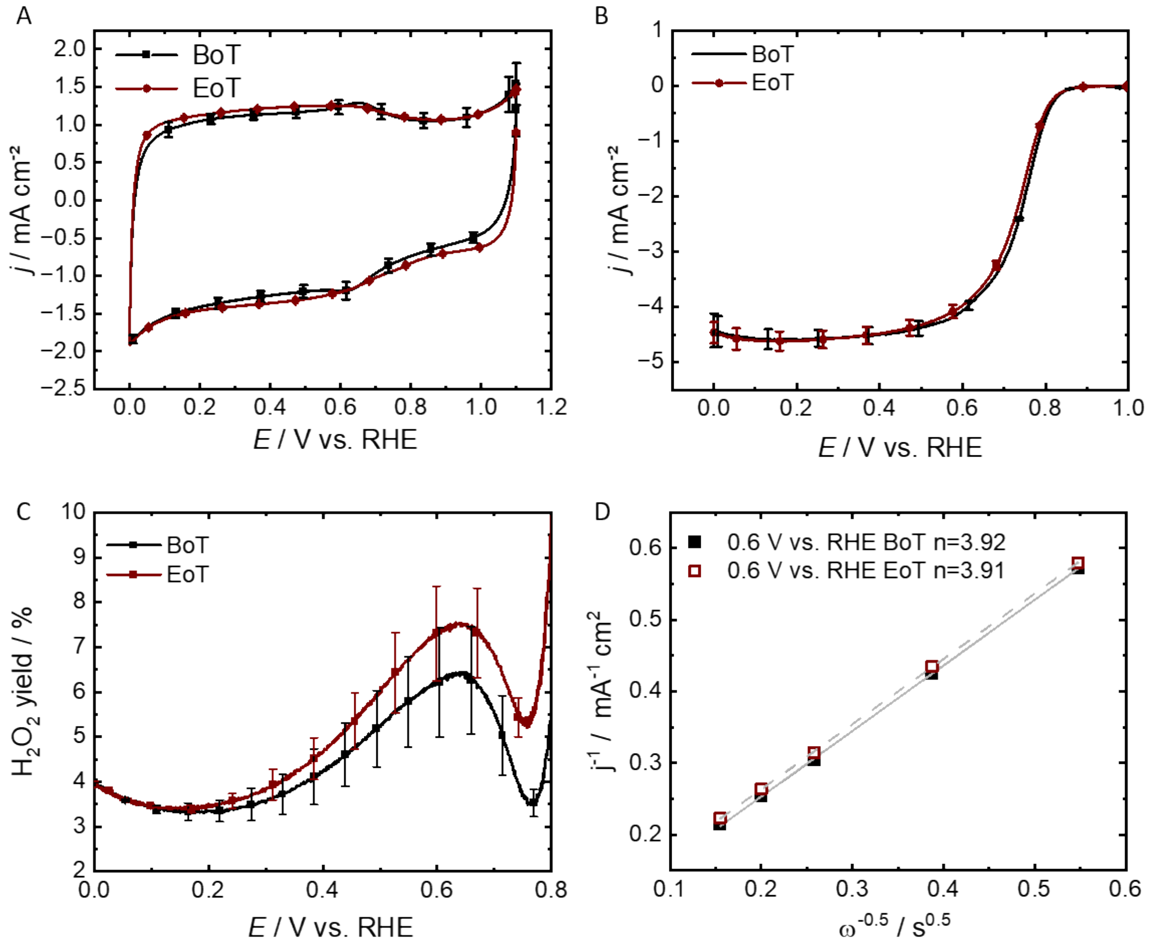

Figure S3. Electrochemistry of FeNC electrodes (+air) at the Beginning of test (BoT) and End of test (EoT) of the SEC-Mössbauer protocol (last step at 0.6 V) in a standard electrochemical setup and in N_2 saturated 0.1 M H_2SO_4. (A) Cyclic voltammograms at scan rate of 10 mV s^{-1}, (B) Linear sweep voltammograms and (C) H_2O_2 yield of FeNC electrode by fix a constant potential of 1.2 V vs RHE at the ring disk with a catalyst loading of 0.51 mg cm^{-2} at BoT and EoT condition (D) Koutecky-Levich plot to estimate the number of transferred electrons at 0.6 V BoT and EoT condition.

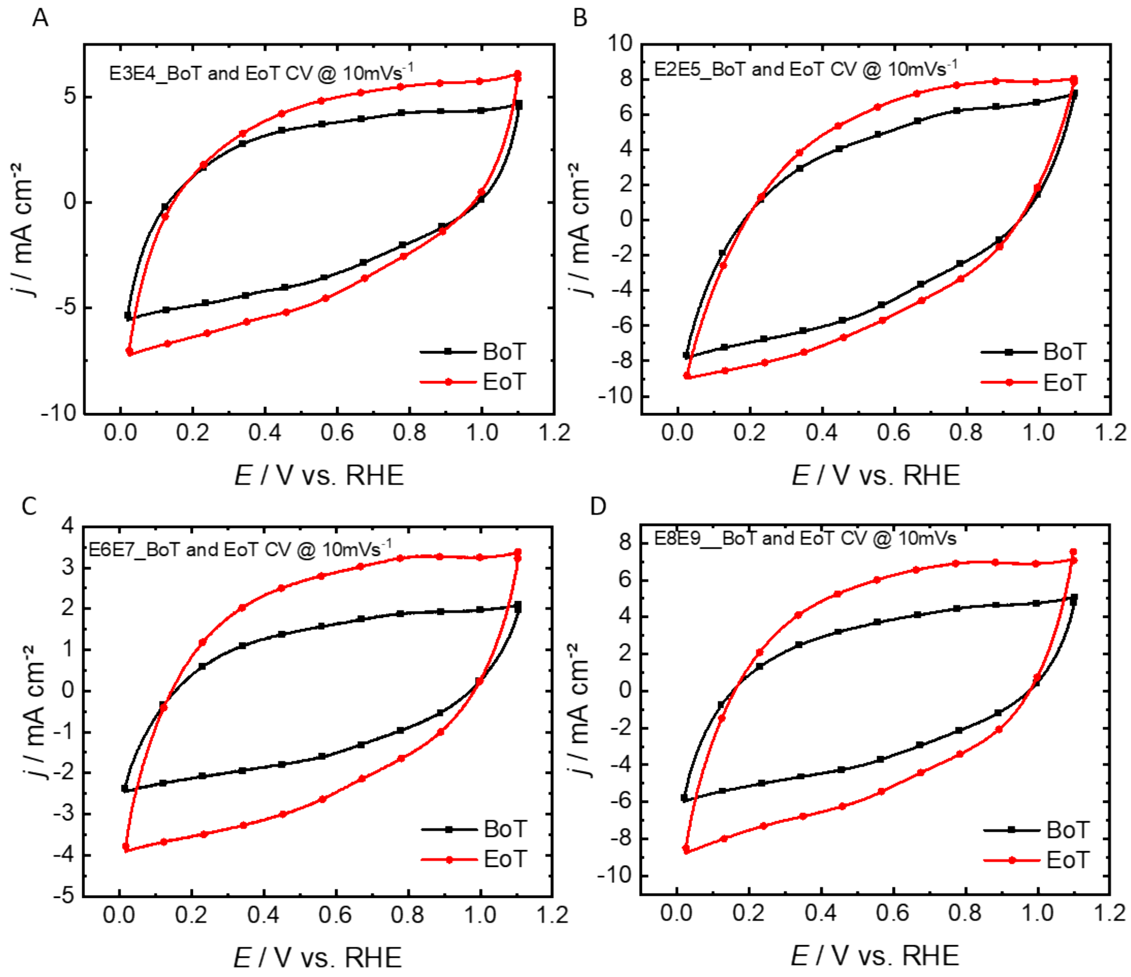

Figure S4. Comparison of cyclic voltammetry curves in SEC-Mössbauer of the four electrodes at BoT and EoT under N_2 saturated 0.1 M H_2SO_4 electrolyte at 10 mV s^{-1} scan rate measured with electrodes (A) E3E4 (B) E2E5 (C) E6E7 and (D) E8E9.

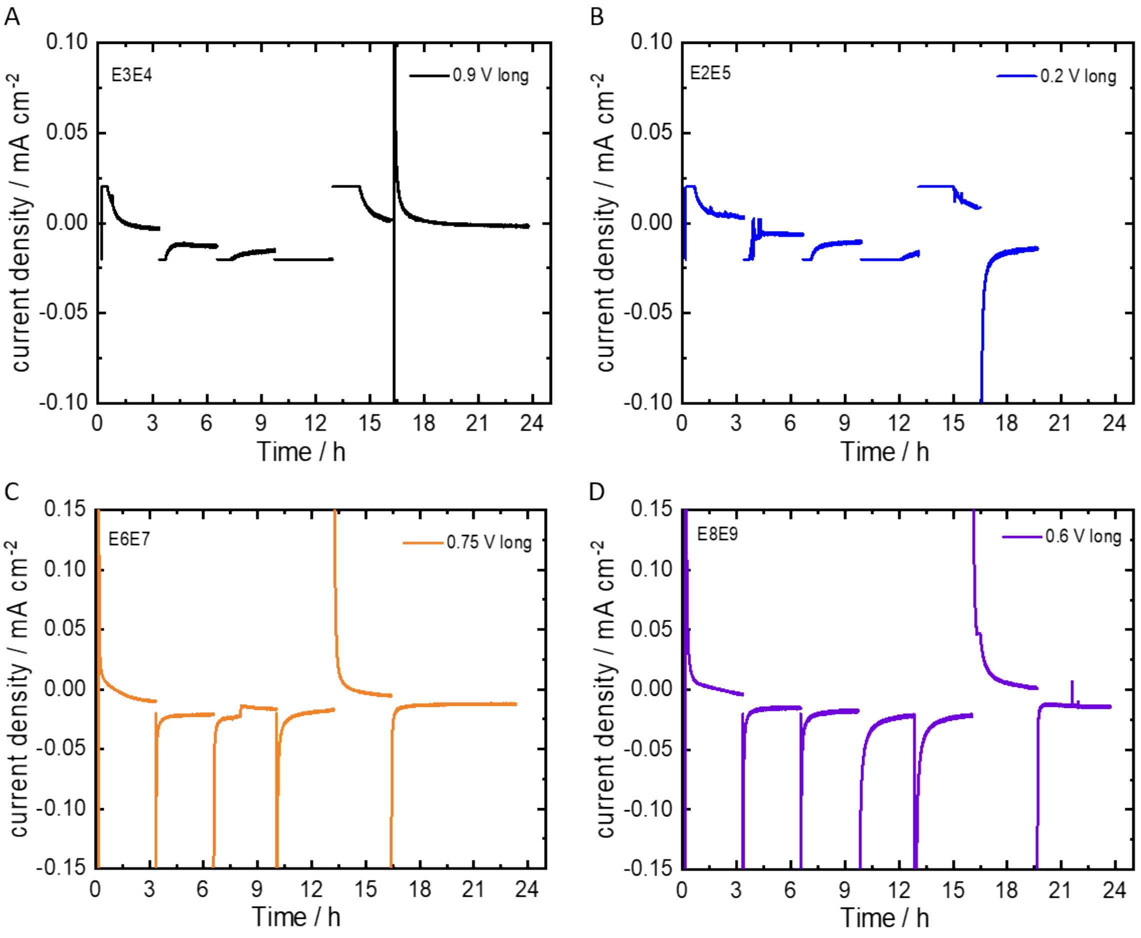

Figure S5. The current density over time for each set of in situ electrodes (SEC-Mössbauer) with long-term tests conducted at (A) 0.9 V, (B) 0.2 V, (C) 0.75 V and (D) 0.6 V.

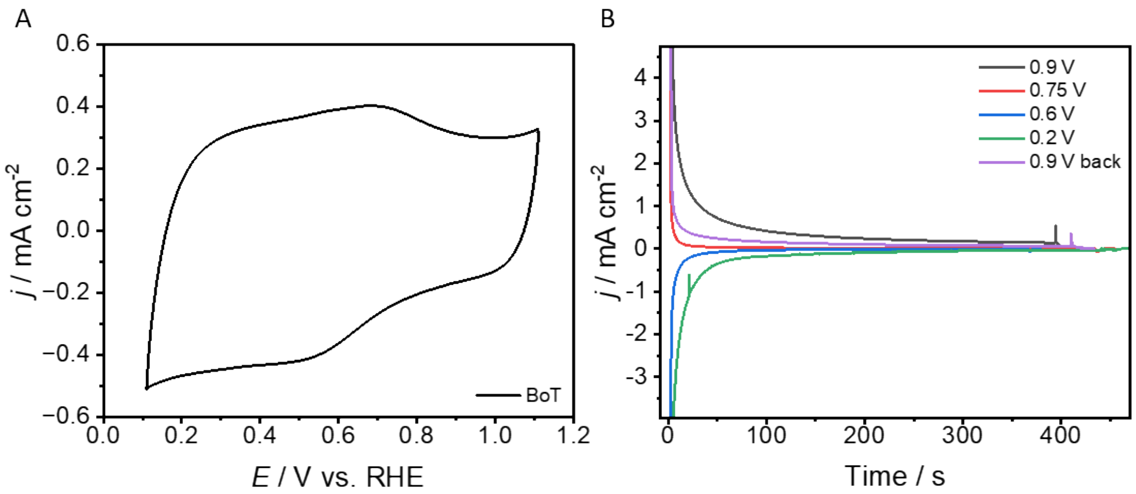

Figure S6. (A) Cyclic voltammetry curves of the FeNC electrode (+air, SEC-EPR) at BoT under N_2 saturated 0.1 M H_2SO_4 electrolyte at 10 mV s^{-1} scan rate. (B) The current density over time for potentials of 0.9 V, 0.75 V, 0.6 V, 0.2 V and 0.9 V_{back}.

3. EPR Spectroscopy

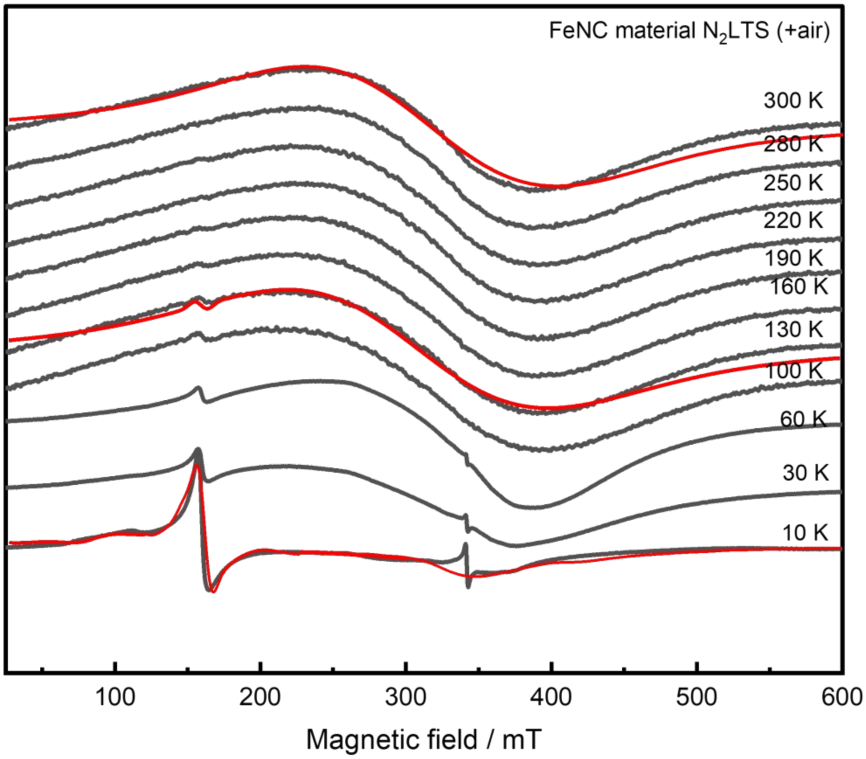

Figure S7. Temperature dependent X-band CW EPR spectra of FeNC after air exposure (N_2LTS +air) in the temperature range from 300 K to 10 K. Experimental spectra (black solid lines) are plotted alongside with an SH (Eq. 1) model assuming the superposition of iron in superparamagnetic structures and isolated rhombic HS Fe(III). Simulation of 300 K, 130 K and 10 K are shown in red.

Figure S7 depicts continuous wave (CW) X-band EPR spectra of FeNC material exposed to air measured between 300 K and 10 K. At 300 K the spectrum is dominated by a several 100 mT broad line centred at $g_{\mathrm{eff}} = 2$. This line further broadened upon decreasing the temperature. Around 200 K an additional resonance appears at $g_{\mathrm{eff}} = 4.3$. Finally, at 10 K the broad line got so broad that it was hardly visible in the CW EPR spectrum, while the EPR resonance with turning points at $g_{\mathrm{eff}} = 10$, $g_{\mathrm{eff}} = 4.3$ and $g_{\mathrm{eff}} = 2$ dominated the CW EPR spectrum. Based on the line shape and the increased linewidth as the temperature is lowered, the broad line was assigned to exchange coupled Fe in superparamagnetic particles. The 10 K spectrum was assigned to isolated high spin (HS, S = 5/2) Fe(III) with small rhombic zero field splitting (ZFS), with assumed axial ZFS $D < 1$ cm^{-1} and $E/D \leq 1/3$. Giving rise to the characteristic resonance at $g_{\mathrm{eff}} = 4.3$. Spectral simulations revealed that the shoulder at $g_{\mathrm{eff}} = 10$ originates from ZFS-strain, which is induced by site-to-site distribution in the Fe(III) coordination.

These broad and narrow contributions to the EPR spectra in Figure S7 were simulated by a superposition two components (para and iso), with the following spin Hamiltonian:

$$\mathcal{H} = \mathcal{H}_{\mathrm{para}} + \mathcal{H}_{\mathrm{iso}}$$

$$\mathcal{H} = \mu_{\mathrm{B}}\boldsymbol{B}_0 \cdot g_1 \cdot \hat{\boldsymbol{S}}_{\mathbf{1}} + \mu_{\mathrm{B}}\boldsymbol{B}_0 \cdot g_2 \cdot \hat{\boldsymbol{S}}_{\mathbf{2}} + D\left[\hat{S}_{2,z}^2 - \frac{1}{3}S_2(S_2+1) + \frac{E}{D}\left(\hat{S}_{2,x}^2 - \hat{S}_{2,y}^2\right)\right] \quad (1)$$

Here the first term, models the contribution of the superparamagnetic iron assuming a fictious spin of S = 1/2 with a *g*-value derived from the zero crossing of the RT EPR spectrum, this resonance was than convoluted with a Voigtian linewidth function. To account for the temperature dependence of this contribution the linewidth and the zero-crossing point of the spectrum were adjusted to the experimental spectrum. The second and the third term model the Zeeman interaction and ZFS contribution of the isolated *S* = 5/2 Fe(III), respectively. *D* and *E* are the axial and rhombic components of the ZFS, respectively.

To simulate the X-band and the Q-band spectra (see Figure S8) of this contribution, very large distribution in the ZFS parameters had to be used. Since the regular perturbation approach implemented in EasySpin[4] (*D*,*E*-strain) did not lead to satisfactory results, a different approach was employed where the explicit accumulation of a large number of simulated spectra with varying *D* and *E* parameters was performed.[9] To exploit the functionality of EasySpin, we used the toolbox to generate a library of X- and Q-band spectra, covering a range of *D* and *E*/*D* values (*D* = 0.05 to 1.0 cm^{-1} and *E*/*D* = 0 to 0.4). This library was then used in a private version of the EasySpin program "pepper", to simulate S = 5/2 spectra with arbitrary distribution in *D* and *E*/*D* parameters by the accumulation of library spectra with appropriate weight distribution and application a variable linewidth to the resulting stick spectrum (generated EPR spectra). To reduce the number of fitting parameters, a simple Gaussian distribution was assumed for both *D* and *E*/*D*. The private version of "pepper" was then used in the EasySpin function "esfit" to optimize the simulation of the FeNC spectra using 5 fitting parameters: (*D*0 = central value of D; dD = Gaussian width of D distribution; ED0 = central value of E/D; dED = Gaussian width of the E/D distribution; LW = Gaussian linewidth). The fitting of X-band and Q-band spectra was found to be heavily dependent on the magnitude of *D*. A Using the following parameters; *D*0 = 0.4 cm^{-1}; dD = 0.47 cm^{-1}; ED0 = 0.24 cm^{-1}; dED = 0.29 cm^{-1}; LW = 10.5 mT, resulted in good agreement for the g_{eff} = 4.3 and g_{eff} = 10 regions of the X-band spectrum (Figure 8A and 8B) and the overall shape of the Q-band spectrum. However, these parameters led to discrepancies in the g_{eff} = 2 of the X-band spectrum. With a smaller *D* (Figure 8C and 8D) using the following parameters; *D*0 = 0.18 cm^{-1}; dD = 0.96 cm^{-1}; ED0 = 0.17 cm^{-1}; dED = 0.2 cm^{-1}; LW = 6.4 mT, the spectrum in the g_{eff} = 2 region was recovered and a much better simulation was achieved at X-band. However, a worse agreement with the Q-band spectra was obtained. Our fitting approach reproduced the main features of the X-band and Q-band 10 K spectra. The remaining discrepancy between the calculated and simulated X- and Q-band spectra may indicate that the ZFS-strain has an even more complicated distribution function. Nevertheless, our simulations clearly show that the observed HS Fe(III) states exhibit rhombic and strongly distributed ZFS with a *D* significantly smaller than 1 cm^{-1}. Thereby providing important insight in the possible coordination environment of these sites in the FeNC environment. EPR spectral fitting model, script and stick spectra for species with large distribution in zero field parameters E and D such as Fe(III) in FeNC material are found https://doi.org/10.17617/3.WIW7CI.

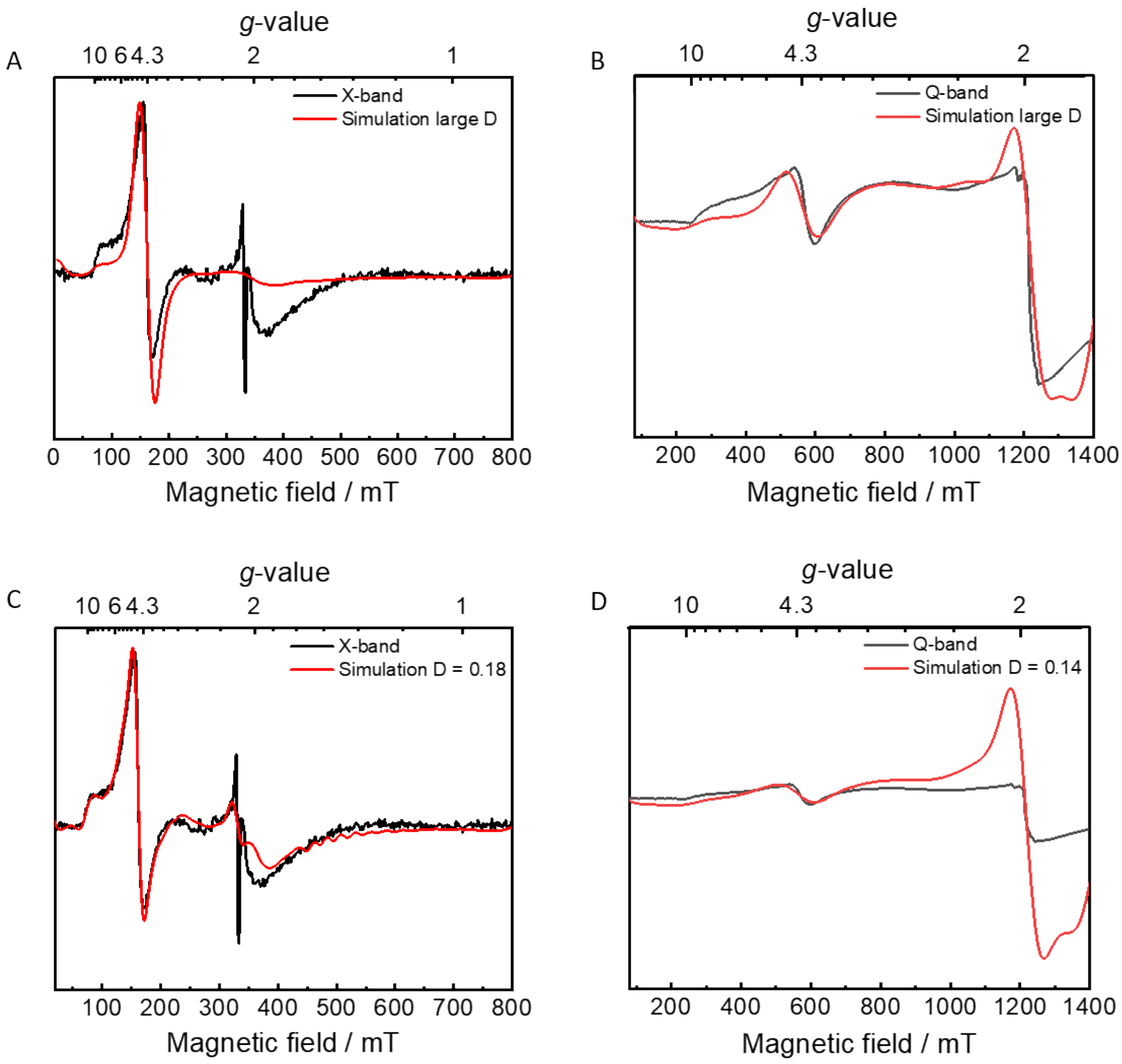

Figure S8. 10 K CW (A) X-band and (B) Q-band EPR spectra of FeNC material (+air, black traces) with simulations (red traces) obtained with Eq. 1 and the simulation routine described in the text with the parameters: $S = 5/2$, $g_{iso} = 2.0$, $D0 = 0.4$ cm^{-1}, dD = 0.47 cm^{-1}, ED0 = 0.24 cm^{-1}, dED = 0.29 cm^{-1}, LW = 10.5 mT. C) and D) identical experimental X- and Q-band CW EPR spectra as in A) and B) alongside simulations obtained with parameters: $S = 5/2$, $g_{iso} = 2.0$, $D0 =$ 0.18 cm^{-1}, dD = 0.96 cm^{-1}, ED0 = 0.17 cm^{-1}, dED = 0.2 cm^{-1}, LW = 6.4 mT. Simulated and experimental spectra are normalised to the sharp signal a $g_{eff} = 4.3$. For small D value. Experimental conditions at X-band: 0.2 mW P_{mw}, 100 kHz modulation frequency and 7 G modulation amplitude and at Q-band: 0.02 mW P_{mw}, 100 kHz modulation frequency and 7 G modulation amplitude.

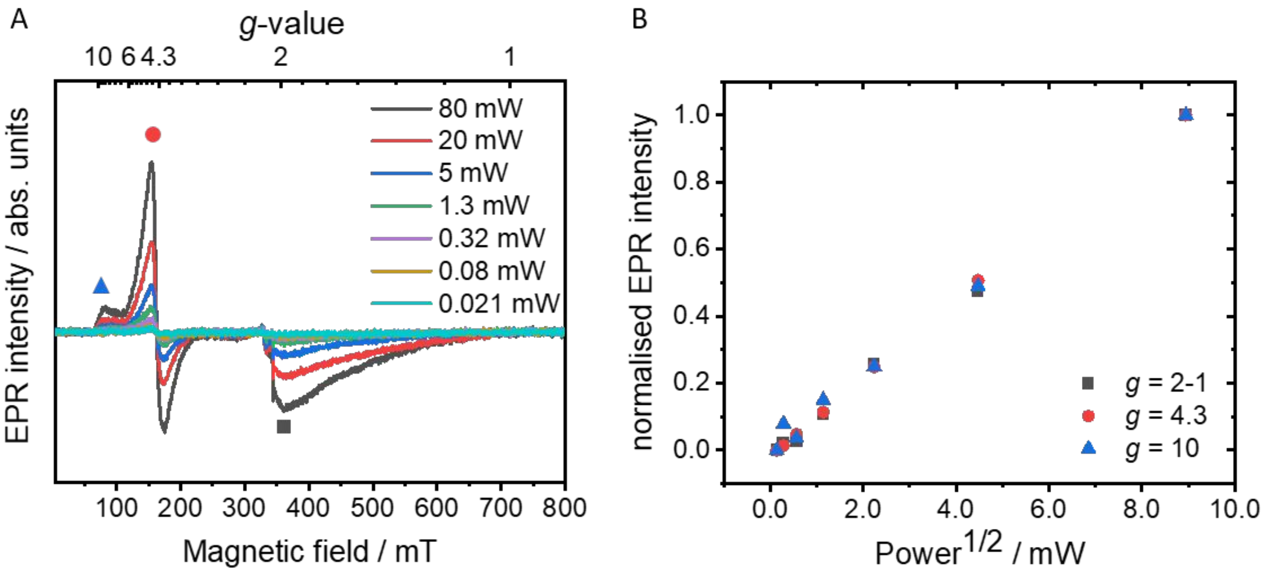

Figure S9. Microwave power (P_{mw}) dependence of CW EPR spectra of FeNC materials (+air). (A) X-band EPR spectra of FeNC materials at 10 K plotted for P_{mw} = 0.02 mW to 80 mW. (B) EPR intensity of three different points (indicated with triangle, circle and square symbols in (A)) in the EPR spectra vs $\sqrt{P_{mw}}$.

4. Mössbauer Spectroscopy

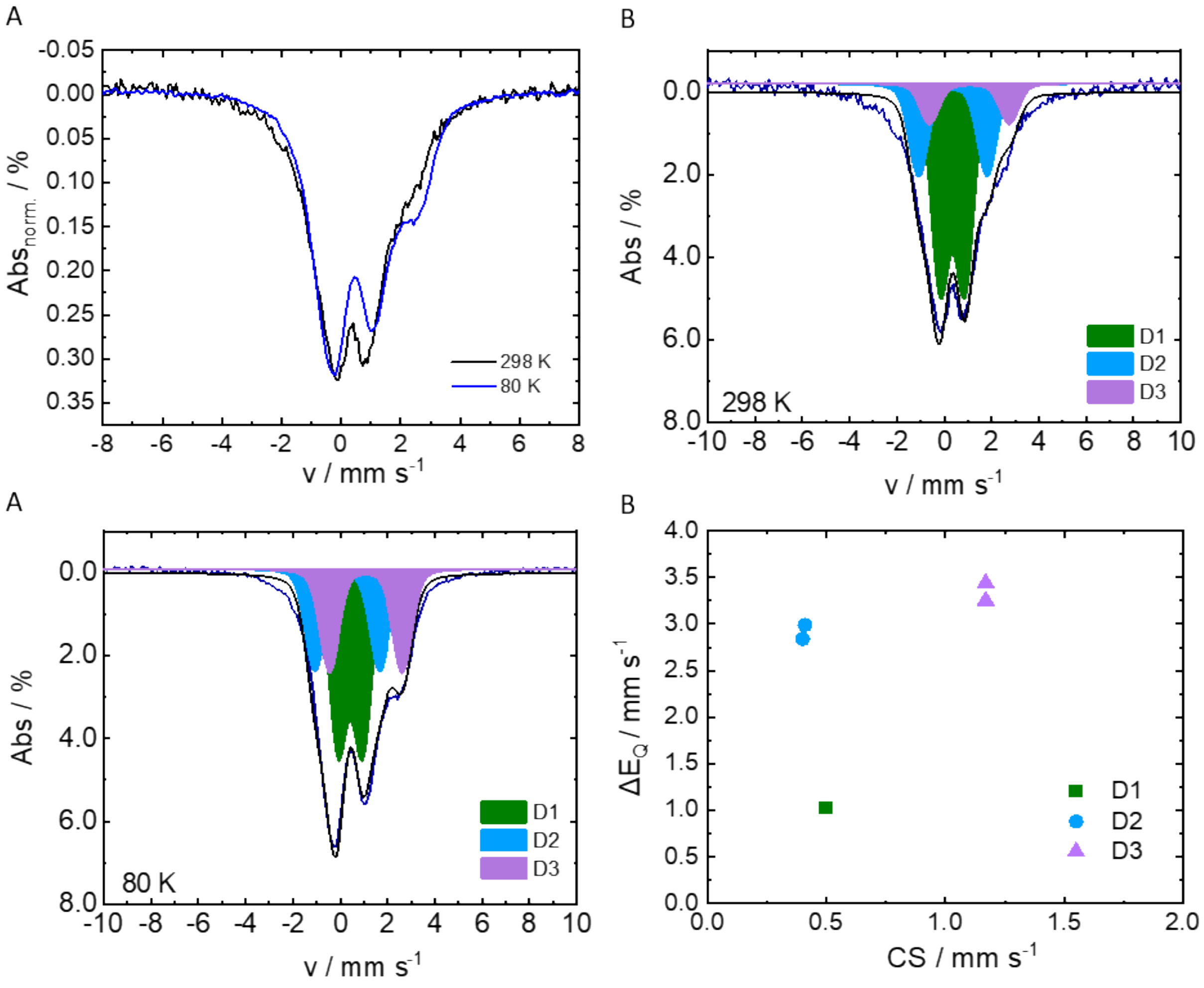

Figure S10. (A) Deconvolution of Mössbauer spectra of FeNC materials (N_2) at 80 K. (B) ΔE_Q vs CS plot of doublets D1, D2 and D3 obtained with Fit model 2.

Depicted in Figure S11 are temperature-dependent Mössbauer spectra (50 K to 1.5 K) of the FeNC materials after air exposure. The spectra exhibited the typical shape encountered in this class of materials, a narrow doublet D1 with CS = 0.48 – 0.50 mm s^{-1} and ΔE_Q = 1.00 – 1.09 mm s^{-1} and additional components which gave rise to shoulders with different intensities at ~ -1 mm s^{-1} and ~ 3 mm s^{-1}. Similar features have been simulated with one or more additional doublets (D2, D3 etc.).[8,10–14] While lowering the temperature to 5 K and below an additional broad component appeared, which was assigned to sextets originating from exchange coupled iron atoms, in e.g. iron oxides. However, the sextet resonance peaks observed in the low-temperature spectra were not fully resolved, which indicated overlay of different sextet contributions. Their assignment was further complicated by the fact that the sextet contribution still increased and further broadened by lowering the temperature from 5 K to 1.5 K. This could indicate that the sextets even at 1.5 K are not fully relaxed, which has been observed previously for iron oxide nanoparticles.[15	,16	]

At all temperatures the Mössbauer spectra exhibited strongly broadened lines. The observed broadening was assumed to at least partly originate from an overlap of different Fe-sites with pronounced site-to-site disorder in the Fe coordination. Likely this originates in the preparation that results in an amorphous carbon that typically exhibits x-ray amorphous behaviour. Such disorder can lead to strain in the single-site static coordination parameters

determining the EPR line shapes (in the present case predominantly *g*- and ZFS-values see Figure 2C, Figure S8) and Mössbauer spectra (CS and ΔE_Q values). The contribution of site-to-site disorder to the linewidth can be modelled by a Gaussian function convoluted with the Lorentzian line shape to achieve the final Voigtian linewidth. Alternatively, though physically less plausible, very fast nuclear relaxation times could lead to strongly broadened Lorentzian lines. Excessive line broadening irrespective of the underlying mechanism, complicates the deconvolution of Mössbauer spectra in different components. This is particularly the case, when the resonance lines of the individual doublets and sextets are only partly resolved in the spectrum like in the present case. In this case, fits of numerical line-shape models to experimental spectra lead to a strong correlation of the obtained parameters and viz uncertainties in the number and characteristics of the Mössbauer components. Under these conditions, robust prior assumptions on the number of different Fe environments, their Mössbauer parameters and line shape are required before adjusting the simulation parameters to the experimental data by numerical fitting. Determining the uncertainties of the obtained fits is not straightforward and therefore different models were tested to estimate these uncertainties.

To test how the different line shape models and relaxation times affect the fits of the spectra, we compared two models (Figure S11). The comparison of the fit models, revealed that at least three doublets and a sextet component (at 5 K and below) are needed to achieve a decent simulation of the temperature-dependent Mössbauer spectra. Adding more doublet components increases an already present correlation of the fitting parameters and reduces the certainty of the information extracted. The three doublets D1, D2 and D3 were assumed with starting parameters determined from the peak positions in the Mössbauer spectra and comparison to literature. In addition, sextet contributions to account for the broad contribution in the low-temperature Mössbauer spectra were added. To account for the broad sextet contribution at very low-temperatures either an overlap of different sextets belonging to the same oxide or a single sextet and partial temperature-dependent averaging of this component was considered. Relaxation in the sextet contribution and the way how the individual line broadenings of the doublets were considered were the main differences between the two fit models described below.

Fit Model 1 was implemented in the Recoil software[7] with a Lorentzian Site Analysis (LSA). In the LSA, the sextets are always considered as fully relaxed and a purely Lorentzian full width at half maximum (FWHM) line width is assumed. The fit employed three doublets (D1–D3) with FWHM of 0.8 mm s^{-1} to ca. 1.06 mm s^{-1} for all doublet components. Additional two sextets were added to the 5 K and 1.5 K spectra, with CS = 0.41 mm s^{-1} (5 K) and CS = 0.48 and 0.56 mm s^{-1} (1.5 K). HF values of 26.8 T and 52.0 T at 5 K and 26.4 T and 48.6 T at 1.5 K were determined for Sext1 and Sext2, respectively. Based on the temperature induced change between 50 K and 5 K it is evident that the sample contains superparamagnetic iron species. Based on preparation we assume a broad distribution of various particle with different relaxation times, based on this large Lorentzian line broadening (1.0 - 2.6 mm s^{-1}) were used. Moreover, it was assumed that both sextets belong to the same species with Sext2 representing superparamagnetic iron environments which are fully magnetically relaxed (or close to it) and Sext1 representing those that are not yet relaxed. The velocity range of ± 10 mm s^{-1} used for the measurement was insufficient to obtain a flat line at the outer edges of the spectra. To correct for this, the zero-absorption line was manually adjusted, as the fit program's default baseline calculation is based on the average of the outer parts. As a consequence, the contribution of the sextet species may be underestimated. The spectra using Fit model 1 are given in Figure S11 left, and the MS parameters are listed in Table S3.

Fit Model 2 was implemented using MF2 Zero-field Mössbauer spectra written by Eckhard Bill[6] with the same number of doublets as in Fit model 1, but a Voigtian line-width model with a 0.2 - 0.3 mm s^{-1} Lorentzian and a 0.7 - 0.8 mm s^{-1} Gaussian contribution. We took information on distribution and lineshapes of the system determined from EPR to guide the fitting of Mössbauer data with Gaussian lines. In addition, a single sextet contribution was added. Due to the temperature dependence of the Mössbauer spectra, this contribution was assumed to be not fully relaxed even at cryogenic temperatures. This scenario was modelled with the following Mössbauer parameters: CS = 0.5 mm s^{-1} and HF = 46 T at 5 K and 49.8 T at 1.5 K and temperature dependent relaxation times of 2.0 x 10^{-9} s^{-1} at 5 K and 3.3 x 10^{-9} s^{-1} at 1.5 K, respectively (Figure S11 right, Table S3).

Comparison of the relative abundances of each Mössbauer component revealed that both models reproduced the general trends of the temperature dependence in the FeNC spectra. However, Fit model 1 and 2 determined significant differences in the absolute contributions of the various components to the spectra. For fit model 1, as

expected with decreasing temperature the amount of D1 was significantly reduced. Starting from 78 % at 50 K, 45 % are found at 5 K and only 28 % at 1.5 K. The decrease in D1 population is mainly due to the formation of sextets. Lowering the temperature to 5 K and 1.5 K enabled magnetic ordering of increasing fractions of iron oxides in the system. The sum of D1 plus sextets equals 74% at both 5 K and 1.5 K. Component D2 increased in intensity with decreasing temperature, the opposite trend was observed for D3. Nonetheless, considering the error margin of ca. 3%, they can be assumed as almost constant. CS and ΔE_Q were allowed to change for each of the fittings. For D1, CS remains constant and ΔE_Q marginally decreased. For D2, CS is lower at 5 K and 1.5 K compared to 50 K, however this is within the error margin. ΔE_Q decreased from 3.1 mm s^{-1} to 2.2 mm s^{-1} for D2. For D3, the CS remains constant, while the ΔE_Q decreased. Usually, for well-defined macrocyclic compounds either a constant or an increase of ΔE_Q is expected as the temperature is lowered. Eventually, additional contribution of magnetic species causes a misleading set of parameters, but this is hard to address. Due to the broad line width of the individual components and the unknown temperature-dependent shift of the sextet peaks due to their relaxation, we have refrained from assigning individual components that contribute to the sextet spectrum. The sextet contribution can be fitted assuming additional sextets (see Figure S11E), but this does not lead to more robust assignments.

In Fit model 2, the CS and ΔE_Q values were fixed or kept close to the values obtained at 50 K, independent from the expected change in CS caused by the second order Doppler shift. Also, here, the intensity of D1 decreased from 65 % to 40 % and further 30% at 5 K and 1.5 K, respectively. At 5 K, 33 % are attributed to the Sext1 component and at 1.5 K it is 44 %. This sextet contributions were assumed to collapse to a doublet with Mössbauer parameters in the range of D1. Considering the sum of D1 plus Sext1 gives a slight increase in contribution from 50 K (65 %) to 5 K (72 %) and 1.5 K (74 %). In this case, both D2 and D3 slightly decrease from 21 % to 17 % at 1.5 K and 15 % to 10% at 1.5 K, respectively. Comparing both fits revealed similar contributions of MS components at 5 K and 1.5 K. However, at 50 K, larger differences were observed. The absorption area of D1 is larger in Fit model 1 than in Fit model 2 by 12%, whereas the opposite is observed for D2 with similar difference. D3 was observed to be similar within the error margin between the two fit models. Overall, there is a good agreement in the area distribution for both models. For Fit model 1, the sum of D1 and sextets is almost constant, whereas in case of Fit model 2, the sum is 7% less at 50 K. Fit model 1, however, reflects changes in ΔE_Q values of D2 and D3 that do not match general expectations. The magnetic dispersion and resulting broad variations in relaxation times might have caused this artefact. Here, Fit model 2 seems advantageous as it can address for this. For the further data evaluation Fit Model 2 was employed since it makes more plausible assumptions about the physical origin of line shape parameters of the FeNC materials in terms of their disorder and relaxation properties. It is noted, that Fit model 1 was also tested to simulate SEC-Mössbauer spectra at RT (data not shown) and provided similar trends as for Fit Model 2.

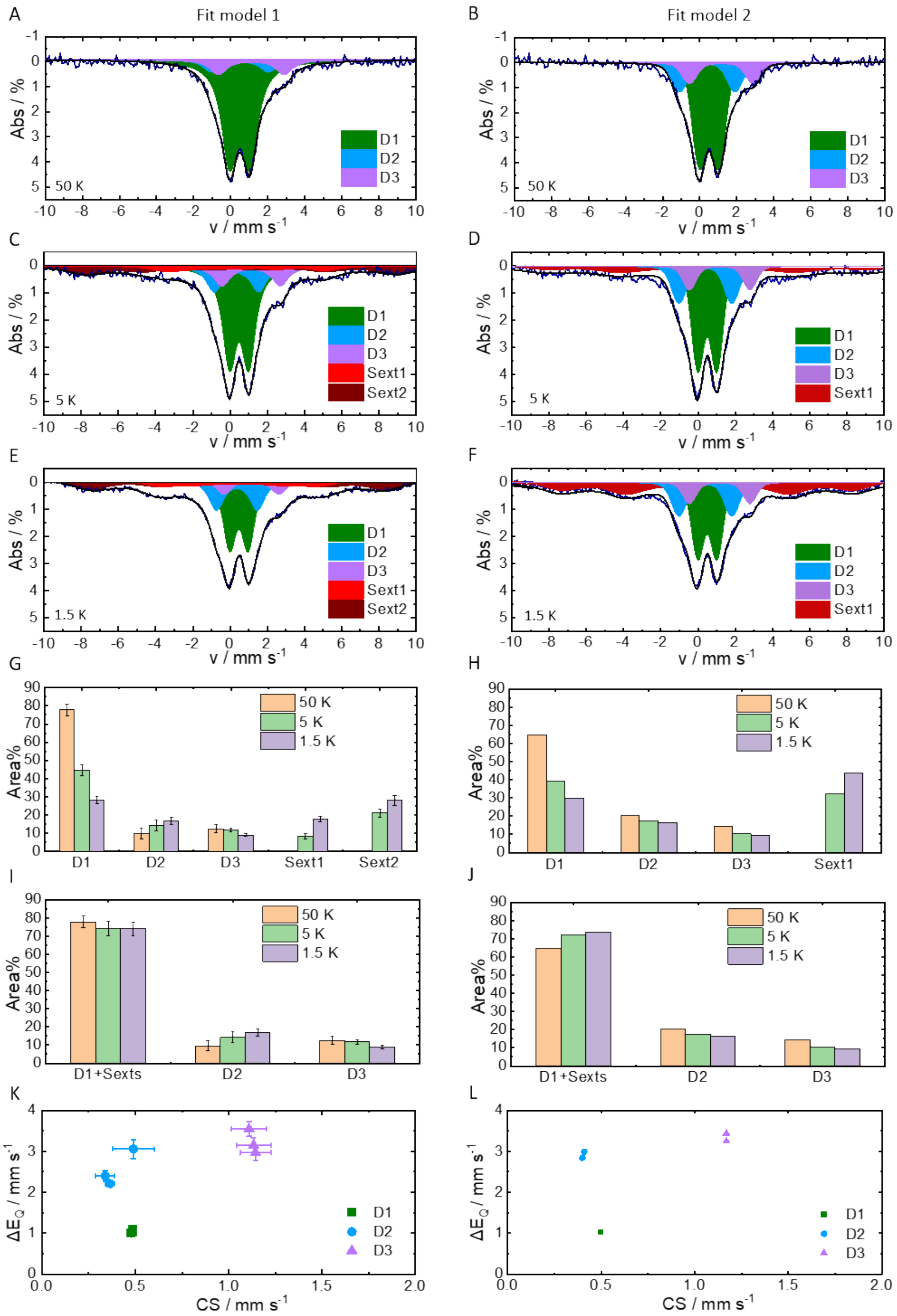

Figure S11. Deconvolution of Mössbauer spectra of FeNC materials (+air) at (A,B) 50 K, (C,D) 5 K and (E,F) 1.5 K with Fit model 1 (left) and Fit model 2 (right). (G,F) Bar graphs of absorption areas of the different Fe species D1, D2, D3 and Sexts present in FeNC material at 50 K, 5 K and 1.5 K obtained with Fit model 1 and 2. (I,J) Different representation of the absorption areas of the different Fe species in (G,F), however D1 and sexts are summed. ΔE_Q vs CS plots of doublets D1, D2 and D3 obtained with (K) Fit model 1 and (L) Fit model 2 for 50 K, 5 K and 1.5 K.

5. Microscopy

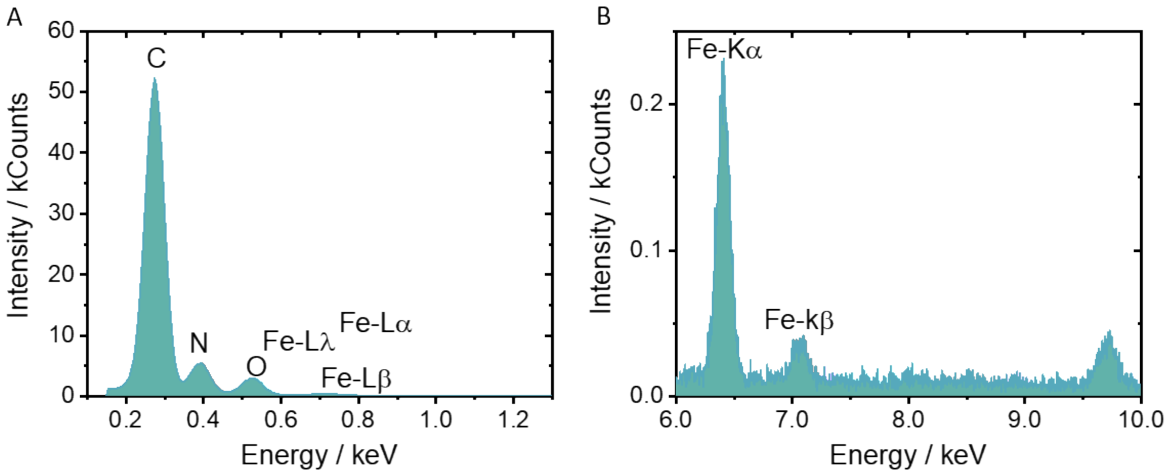

Figure S12. Energy Dispersive X-ray (EDX) spectroscopy of FeNC catalysts in N_2 environment. (A) EDX of FeNC catalysts in N_2 between 0 and 1.3 keV. (B) Extension of the EDX spectrum of the same sample to the range between 6.0 and 10.0 keV.

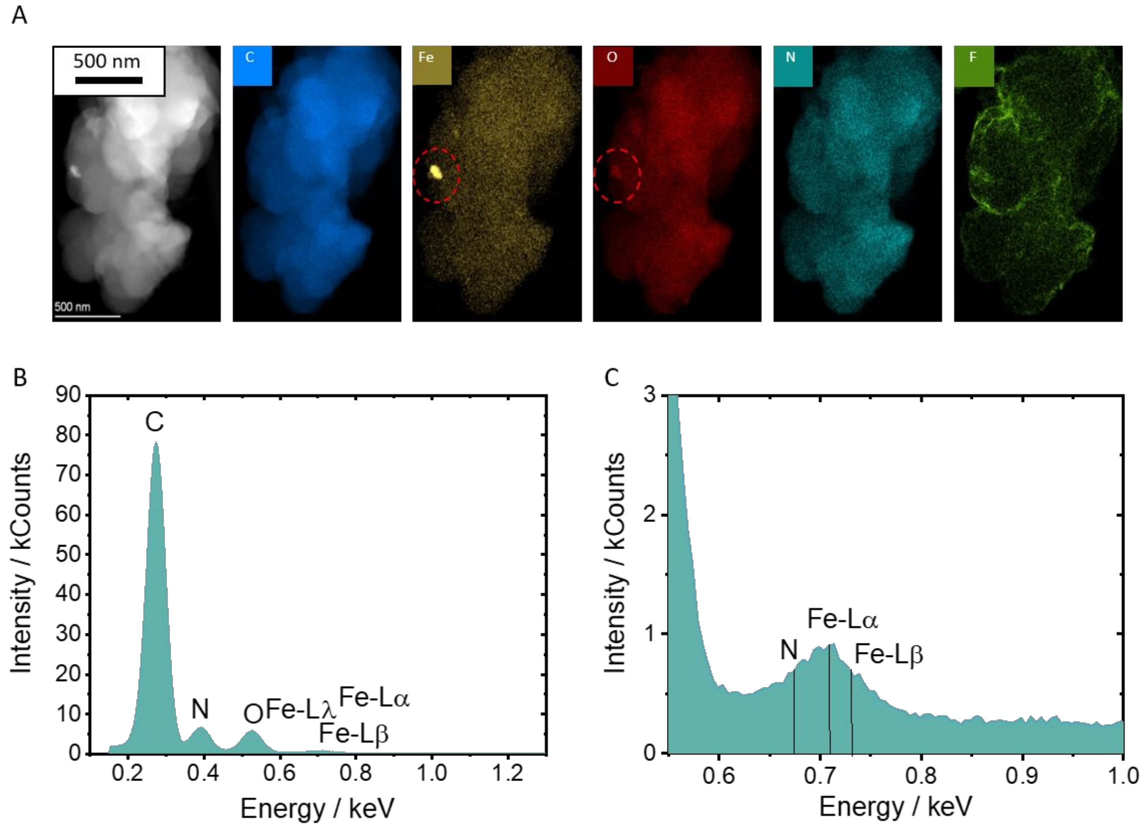

Figure S13. (A) TEM/STEM images of FeNC (+air) prepared as an ink using water, ethanol and Nafion as a binder with EDX elemental mapping of carbon, iron, oxygen, nitrogen and fluorine. Red dashed circle shows iron oxide nanoparticles. (B) EDX spectrum of FeNC ink. (C) Zoom of the EDX spectrum of the same sample as (A) and (B) between 0.5 and 1.0 keV.

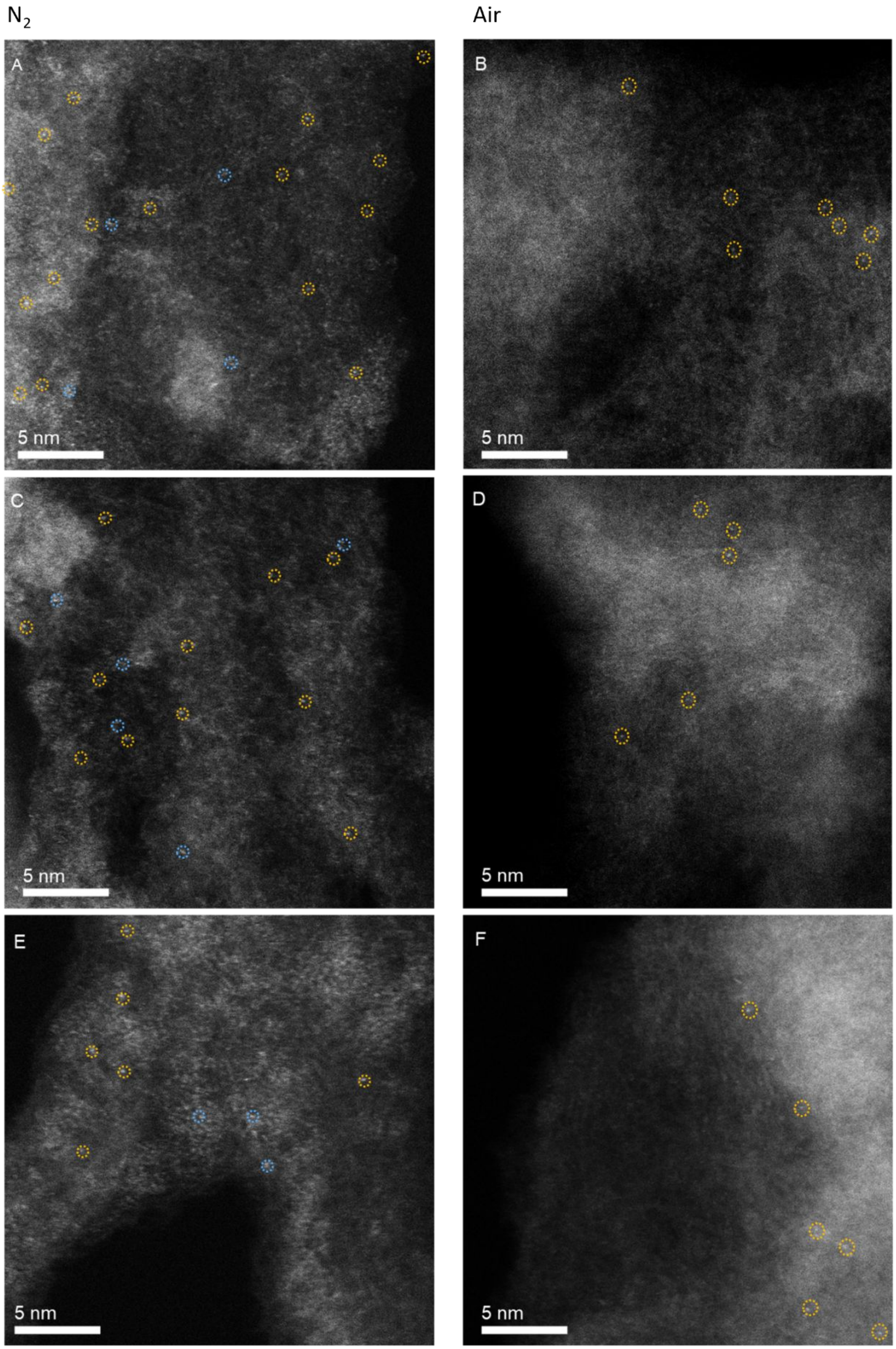

Figure S14. ADF-HRSTEM images of FeNC material (A), (C) and (E) under N_2 and (B), (D) and (F) under +air conditions. Encircled are regions (circle diameter 0.5 nm) where a single Fe (yellow dashed circle) or more than one Fe (blue dashed circle) was identified. The original data for can be found in https://doi.org/10.17617/3.WIW7CI.

6. SEC-Mössbauer Spectroscopy

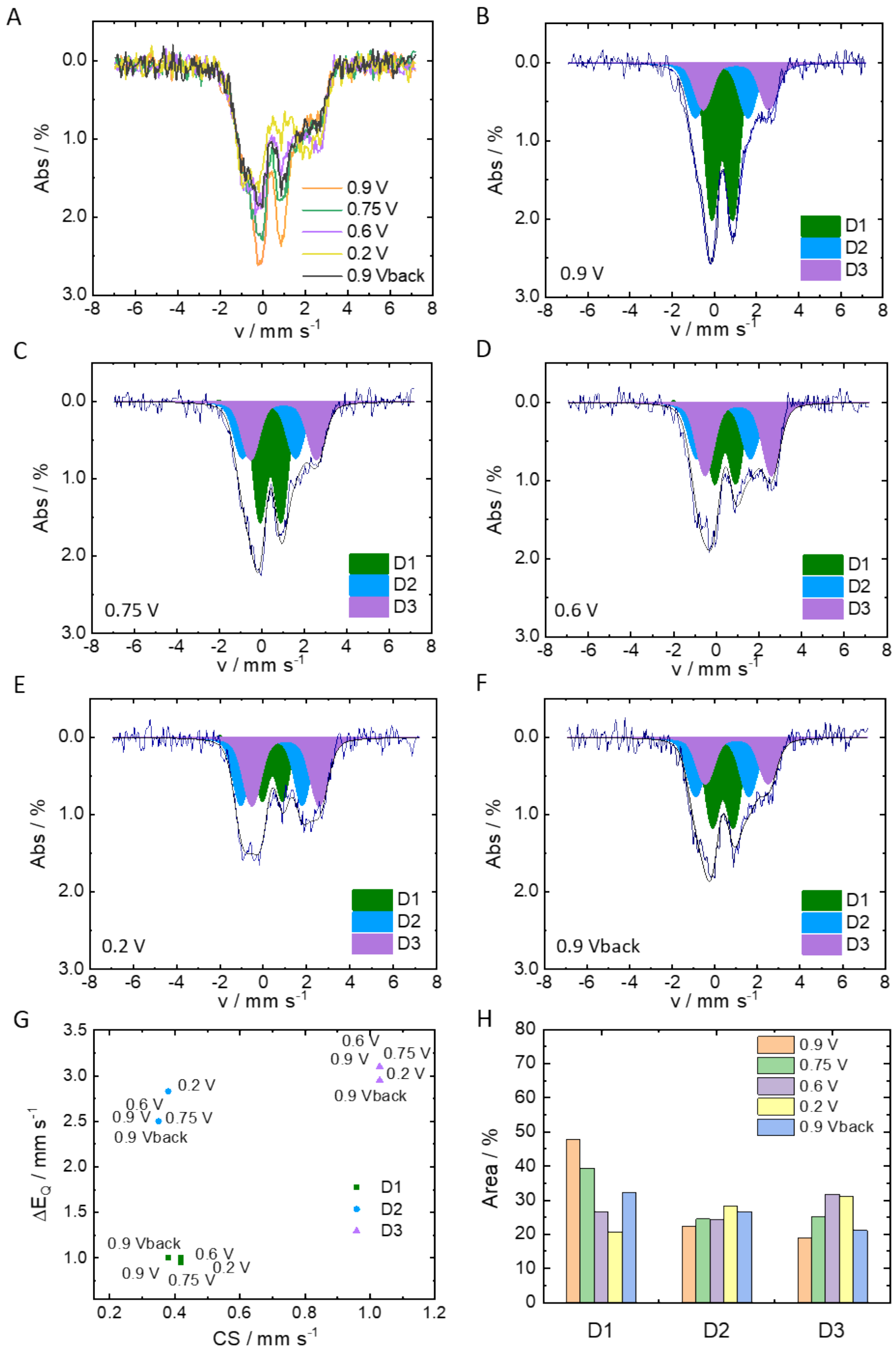

Figure S15. (A) *In situ* RT SEC-Mössbauer spectra of an FeNC electrode (+air) in N_2 saturated electrolyte at potentials of 0.9 V (orange), 0.75 V (green), 0.6 V (purple), 0.2 V (dark yellow) and 0.9 V back (blue). SEC-Mössbauer spectra overlaid with simulations obtained with Fit model 2 at (B) 0.9 V, (C) 0.75 V, (D) 0.6 V, (E) 0.2 V and (F) 0.9 Vback. (G) Comparison of CS and ΔE_Q of the doublets D1, D2 and D3 in the FeNC electrode obtained from SEC-Mössbauer measurements. (H) Bar graph of the doublet contributions (leaching corrected) as a function of potential.

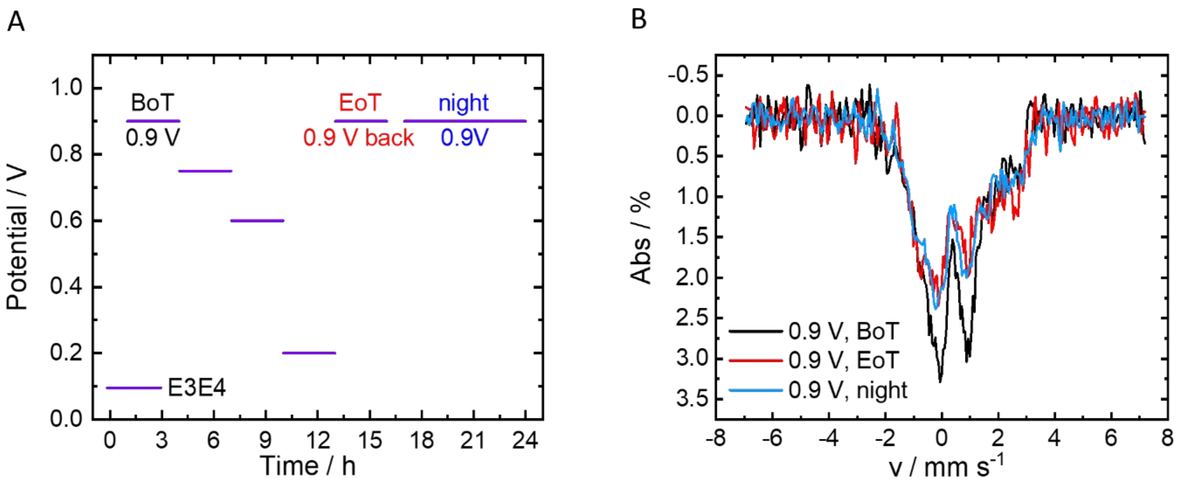

Figure S16. A) In situ Mössbauer measurement protocols with BoT, EoT and night highlighted for electrode E3E4. B) RT SEC-Mössbauer of +air FeNC electrode in N_2 saturated electrolyte at 0.9 V measured at the BoT (black), EoT (red) and at night (blue). The respective times at which the measurements were recorded during the measurement cycle are shown in A.

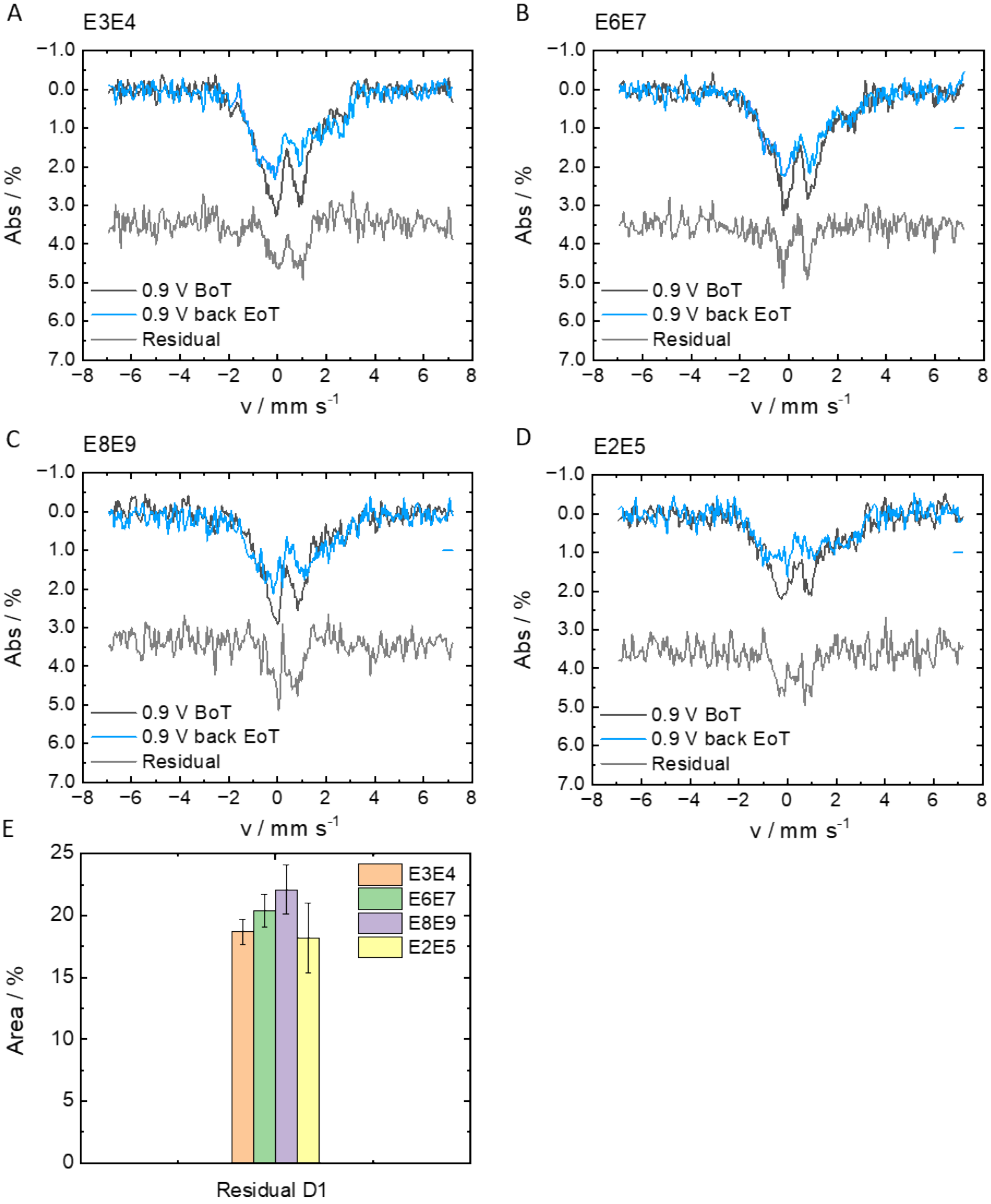

Figure S17. RT SEC-Mössbauer of (+air) FeNC electrodes measured at 0.9 V (BoT, black) and at 0.9 Vback (blue) for different electrodes: A) E3E4, B) E6E7, C) E8E9 and D) E2E5. In grey are shown the differences of spectra taken during leaching and for saturated leaching, plotted against an offset 3.5 abs % for better visibility. The spectra were baseline correct using a linear correction. E) Residual integrated area as a percentage of the total integrated area at 0.9 V (BoT). The average leaching observed for all four electrodes is 19 ± 3%.

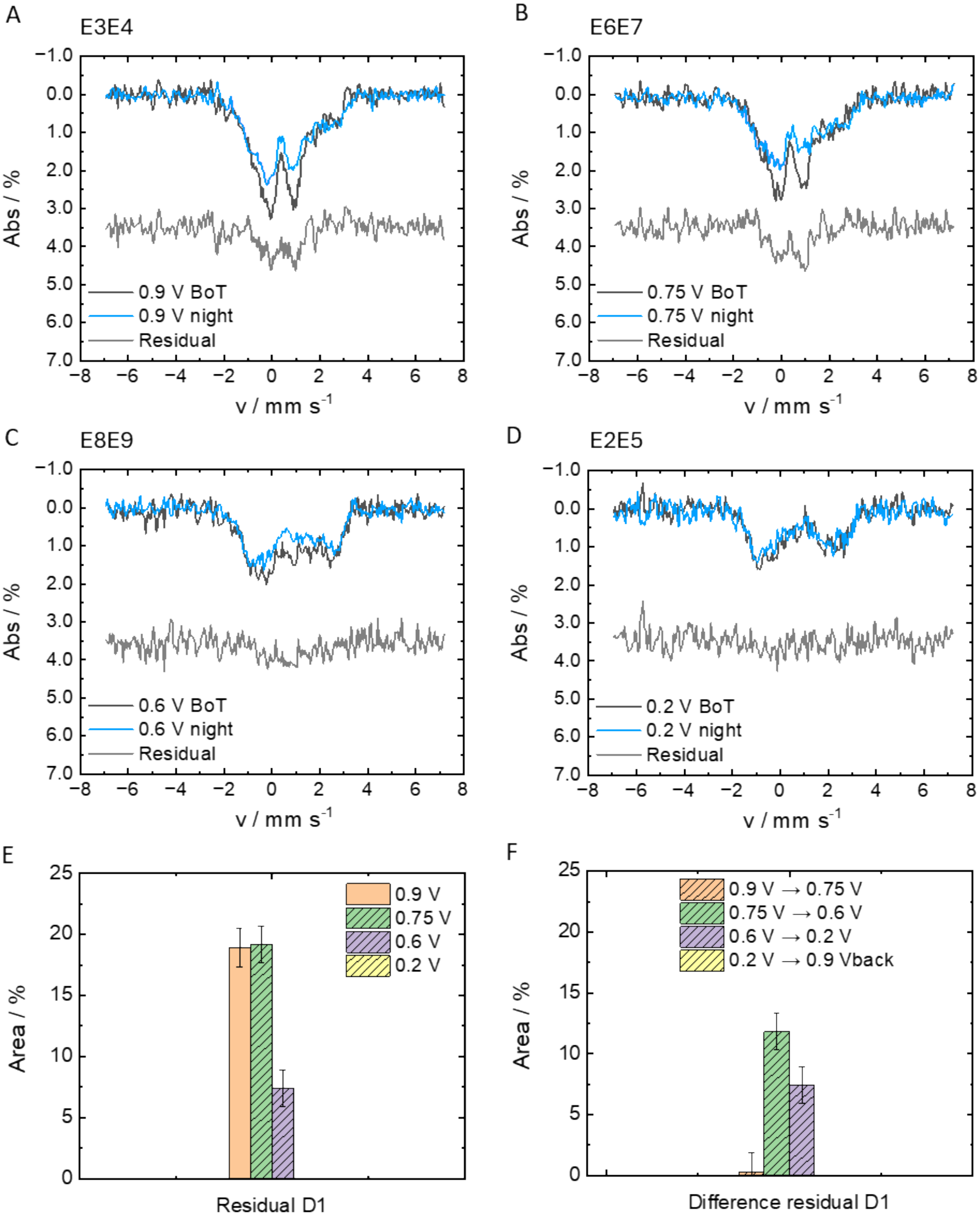

Figure S18. RT SEC-Mössbauer of (+air) FeNC electrodes measured at the beginning of testing (BoT, black) and for saturated leaching (night, blue) at A) 0.9 V, B) 0.75 V, C) 0.6 V and D) 0.2 V. The spectra were baseline correct using a linear correction. After applying a potential of 0.2 V no further significant leaching is observed. In grey are shown the differences of spectra taken at BoT and night, plotted against an offset 3.5 abs % for better visibility. E) Residual integrated area of D1 as a percentage of the initial unleached spectra (BoT, black). F) Difference in the residual integrated area of D1 between subsequent potential steps.

Leaching correction

The Mössbauer spectra of the FeNC electrodes in Figure S17, all of which were recorded at 0.9 V on the identical electrode before and after the potential cycle, show that leaching primarily affects D1. On the other hand, the spectra shown in Figure S18, which were recorded at different potentials before (BoT) and after (night) the potential cycle, show that the spectral changes caused by leaching can no longer be detected within the S/N of the experiment once the electrode has completed the cycle from 0.9 V to 0.2 V. Based on these findings, we independently determined the contribution of leaching to the RT-SEC Mössbauer spectra (Figure 6 and S15).

From spectra shown in S17 the amount of leaching of a full potential cycle for all four electrodes was obtained individually by evaluating the total loss of the integrated area of the baseline corrected spectrum through leaching. We found the amount of leaching for all for electrodes was comparable and amounted to an average value of 19 ± 3%. After this we obtained the amount of leaching at each potential step from the differences of the integrated spectral areas before and after leaching. These values are given in Figure 18E and 18F. Having determined the leaching at each potential step, we were able to the subtract this contribution from the total redox induced change in the SEC-Mössbauer spectra. We did this by normalizing all observed changes in D1, D2 and D3 from the potential dependent SEC-Mössbauer spectra in Figure S15 to the unleached 0.9 V spectrum obtained at the beginning of testing (BoT, Figure S16A). Since the spectra summarised in Figure S15 were recorded at different time points in the potential cycle, they were weighted accordingly during the leaching correction. The obtained potential dependent contributions of D1, D2 and D3 are given in Figure 7C).

7. Comparison of Mössbauer parameters (CS and ΔE_Q) to literature

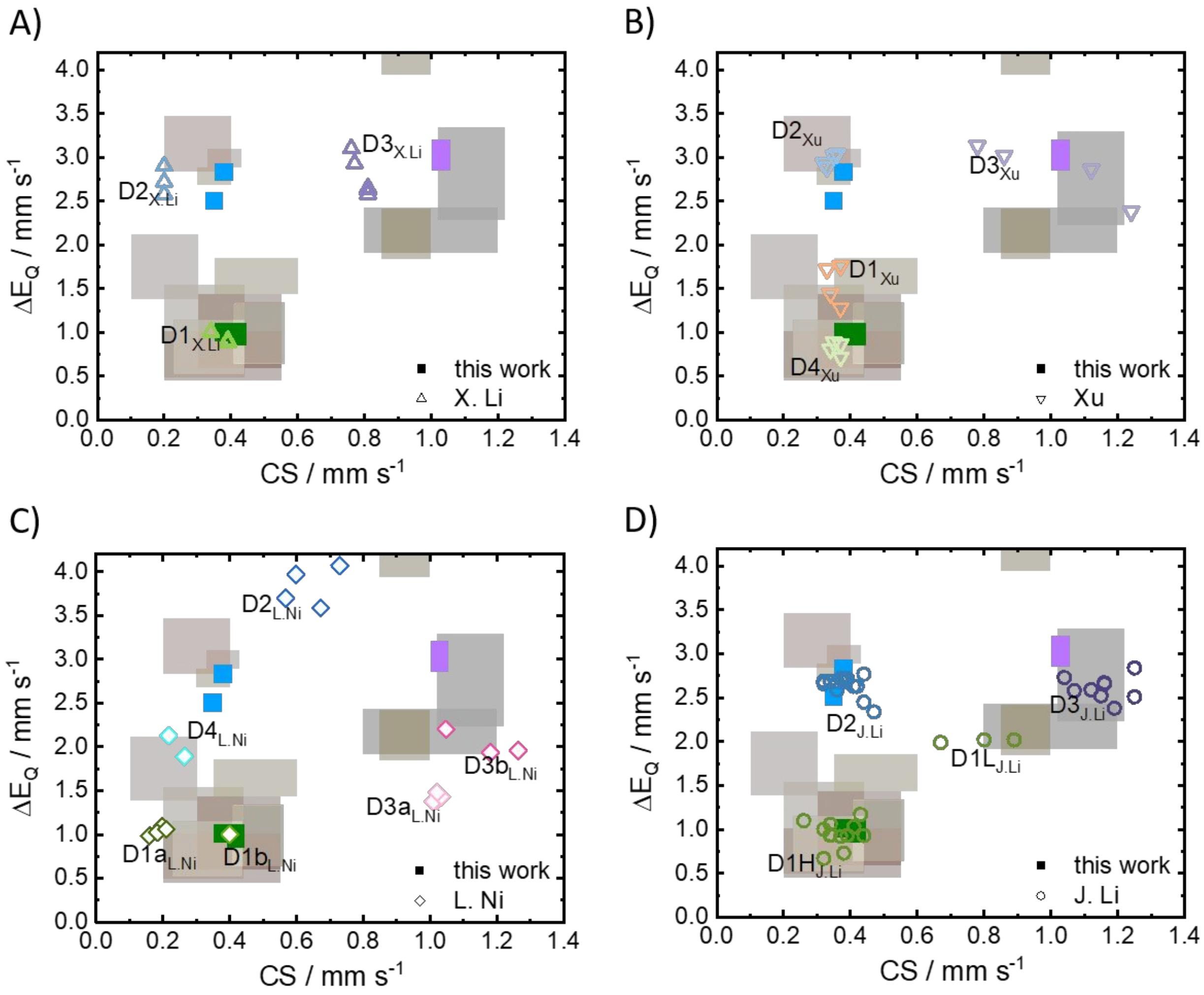

Figure S19. Comparison of CS and ΔE_Q values of D1, D2 and D3 obtained from *in situ* SEC-Mössbauer spectroscopy (our work using Fit model 2 represented by boxes) to values reported for Fe(II) and Fe(III) in FeN_4 macrocycles[10] at RT and iron oxides clusters in glasses.[15,16] The following references were used in (A) X. Li[14] (triangle), (B) Xu[13] (inverted triangle), (C) L. Ni[8] (diamond), (D) J. Li[12] (triangle). The grey boxes represent the range in which HS Fe(II) and HS Fe(III) in iron oxides in glasses are found. The ▪ box represents LS Fe (II) ▪ box represents IMS Fe (II) ▪ box represents HS Fe (II) ▪ box represents LS Fe (III) ▪ box represents IMS Fe (III) ▪ box represents HS Fe (III).

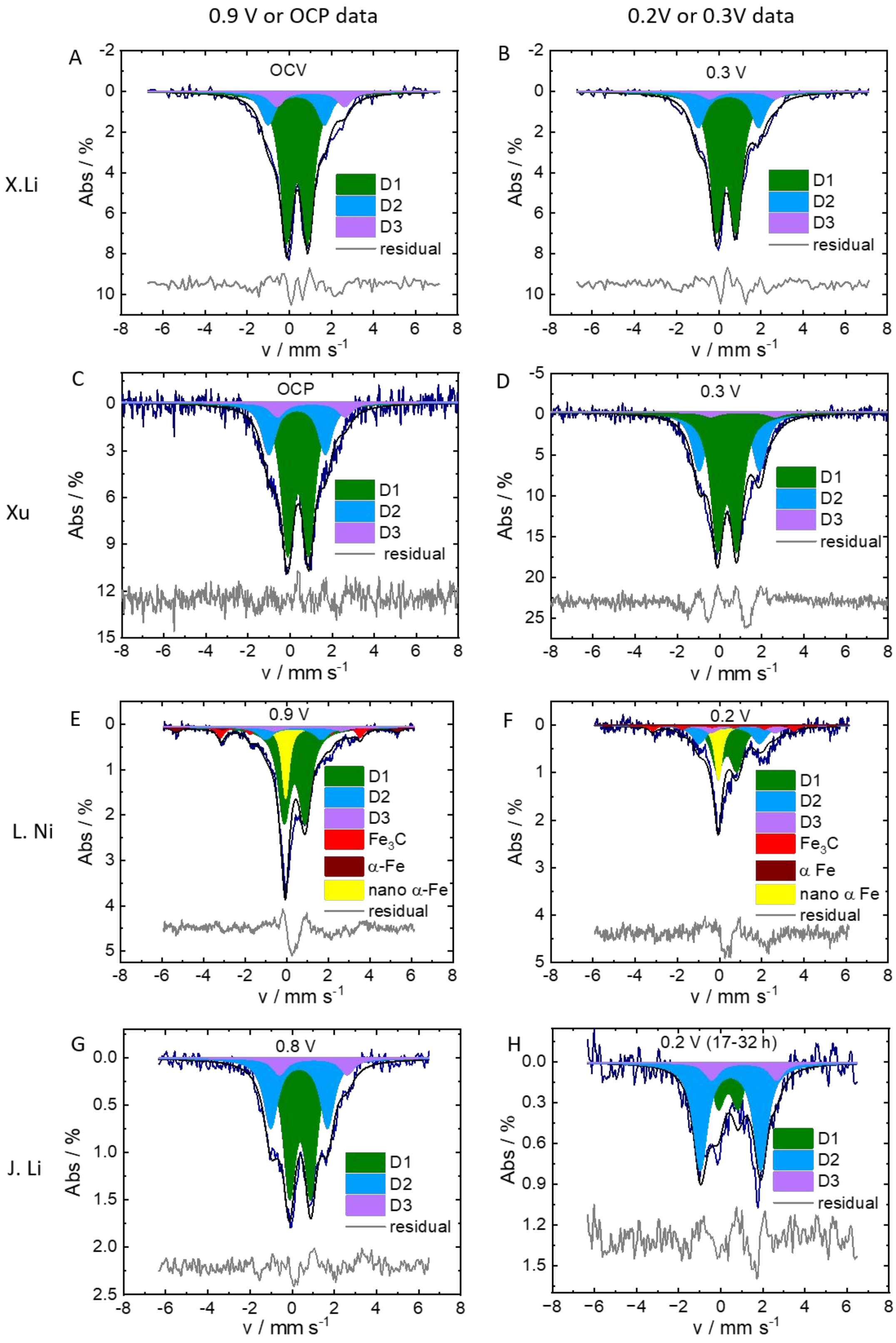

Figure S20. Fits of literature SEC-Mössbauer data (A,B) X. Li[14], (C,D) Xu[13], (E,F), L. Ni[8] (G,H) J. Li[12] at either 0.9 V or OCP (left) vs 0.2 V (or 0.3 V) (right) by Fit model 1, with the D1, D2 and D3 contributions identified in this work.

Table S1. Electrochemistry of FeNC electrode at the Bot and Eot in a standard electrochemical setup mimicking the *in situ* MS protocol of cycling potential steps with the final step being performed at 0.8 V and then U = 0.6 V (RHE). Summary of the electrocatalytic performance data of the FeNC electrode using a catalyst loading of 0.51 mg cm^{-2} in 0.1 M H_2SO_4 and a rotation speed of 1500 rpm.

sample	E_{onset} / V	j_{diff} / mA cm^{-2}	half wave potential $E_{1/2}$ / V	j_{kin} @ 0.8 V / mA cm^{-2}	n (e$^-$)	Selectivity H_2O_2 at 0.6V / %
BoT	0.845	-4.590	0.741	0.672	3.92	6.3
EoT	0.842	-4.629	0.728	0.495	3.91	7.5

Table S2. The Mössbauer parameters for FeNC material as prepared (N_2), after long term storage in liquid nitrogen (N_2LTS) and exposure to air (air) using Fit model 2.

	CS [mm s^{-1}]	ΔE_Q [mm s^{-1}]	H0 [T]	Lorentzian linewidth	Gaussian linewidth	Relative area [%]	Assignments
**FeNC material 298K- N_2**							
D1	0.36	1.03	-	0.2	0.8	58.0	Fe(III) HS
D2	0.38	2.89	-	0.3	0.8	29.2	Fe(II) IMS
D3	1.08	3.37	-	0.3	0.8	12.8	Fe(II) HS
**FeNC material 80K-N_2**							
D1	0.43	1.03	-	0.2	0.8	44.4	Fe(III) HS
D2	0.38	2.50	-	0.3	0.8	27.7	Fe(II) IMS
D3	1.08	3.03	-	0.3	0.8	28.0	Fe(II) HS
**FeNC material 1.8K-N_2**							
D1	0.53	1.02	-	0.2	0.8	20.5	Fe(III) HS
D2	0.40	2.46	-	0.3	0.8	19.2	Fe(II) IMS
D3	1.07	3.10	-	0.3	0.8	22.2	Fe(II) HS
Sext1	0.50	0*	48.14	0.24	1.00	38.0	Fe_2O_3_1
**FeNC material 80K- N_2 LTS**							
D1	0.43	1.03	-	0.2	0.8	60.6	Fe(III) HS
D2	0.38	2.50	-	0.3	0.8	21.2	Fe(II) IMS
D3	1.08	3.03	-	0.3	0.8	18.1	Fe(II) HS
**FeNC material 1.8K- N_2 LTS**							
D1	0.53	1.02	-	0.2	0.8	27.0	Fe(III) HS
D2	0.40	2.46	-	0.3	0.8	14.4	Fe(II) IMS
D3	1.07	3.10	-	0.3	0.8	13.4	Fe(II) HS
Sext1	0.50	0*	48.14	0.24	1.00	45.2	Fe_2O_3_1
**FeNC material 50K- air**							
D1	0.50	1.02	-	0.2	0.8	64.8	Fe(III) HS
D2	0.41	2.84	-	0.3	0.8	20.6	Fe(II) IMS
D3	1.17	3.44	-	0.3	0.8	14.6	Fe(II) HS
**FeNC material 1.5K- air**							
D1	0.50	1.02	-	0.2	0.8	30	Fe(III) HS
D2	0.40	2.84	-	0.3	0.8	16.6	Fe(II) IMS
D3	1.17	3.25	-	0.3	0.8	9.6	Fe(II) HS
Sext1	0.50	0	49.82	0.24	1.00	43.8	Fe_2O_3_1

Error for the CS and ΔE_Q determined by residual of the fitting in Fit model 2 is between 0.1 - 0.2 mm s^{-1}. Error for the H_0 in Fit model 2 is 0.5 – 1.4 T.

Table S3. Mössbauer parameters for FeNC material (+air) at 50 K, 5 K and 1.5 K using Fit Model 1 and 2.

**Fit model 1**							
	CS [mm s^{-1}]	ΔE_Q [mm s^{-1}]	H_0 [T]	Lorentzian linewidth	Gaussian linewidth	Relative area [%]	Assignments
**FeNC material-air – 50 K**							
D1	0.49 ± 0.01	1.09 ± 0.03		1.02 ± 0.04		77.8 ± 3.2	Fe(III) HS
D2	0.49 ± 0.11	3.06 ± 0.23		1.0*		9.7 ± 2.9	Fe(II) IMS
D3	1.10 ± 0.01	3.55 ± 0.18		1.06*		12.5 ± 2.2	Fe(II) HS
**FeNC material-air – 5 K**							
D1	0.49 ±0.01	1.03 ±0.02		0.86*		44.7 ±3.0	Fe(III) HS
D2	0.34 ±0.05	2.40 ±0.13		1.0*		14.2 ±3.0	Fe(II) IMS
D3	1.13 ± 0.01	3.2 ± 0.2		1.06*		11.8 ± 1.2	Fe(II) HS
Sext1	0.41*	0.2*	26.8 ± 1.1	1.6*		8.2 ±1.5	Fe_2O_3_1
Sext2	0.41*	0.36*	52.0 ± 1.1	2.6 ±0.4	-	21.2 ±2.2	Fe_2O_3_2
**FeNC material -air – 1.5 K**							
D1	0.48 ± 0.01	1.00 ±0.02		0.8		28.4 ±2.0	Fe(III) HS
D2	0.37 ± 0.03	2.21 ±0.08		1.0*		16.7 ± 2.0	Fe(II) IMS
D3	1.14 ± 0.08	2.97 ± 0.19		1.06*		9.0 ± 1.0	Fe(II) HS
Sext1	0.48 ±0.2	0 ± 0.15	26.4 ±1.1	2.6*		17.7 ± 1.6	Fe_2O_3_1
Sext2	0.56 ±0.15	-0.12 ±0.12	48.6 ±0.6	2.6*		28.0 ±2.7	Fe_2O_3_2

*Means the value are fixed; error bar (95% confidence interval) were given behind ±. Chemical shift is corrected by α Fe

**Fit model 2**							
	CS [mm s^{-1}]	ΔE_Q [mm s^{-1}]	H_0 [T]	Lorentzian linewidth	Gaussian linewidth	Relative area [%]	Assignments
**FeNC material -air –50 K**							
D1	0.50	1.02	-	0.2	0.8	64.8	Fe(III) HS
D2	0.41	2.84	-	0.3	0.8	20.6	Fe(II) IMS
D3	1.17	3.44	-	0.3	0.8	14.6	Fe(II) HS
**FeNC material -air –5 K**							
D1	0.50	1.02	-	0.2	0.8	39.5	Fe(III) HS
D2	0.40	2.84	-	0.3	0.8	17.6	Fe(II) IMS
D3	1.17	3.25	-	0.3	0.8	10.3	Fe(II) HS
Sext1	0.50	0	46.0	0.24	1.00	32.6	Fe_2O_3_1
**FeNC material -air –1.5K**							
D1	0.50	1.02	-	0.2	0.8	30	Fe(III) HS
D2	0.40	2.84	-	0.3	0.8	16.6	Fe(II) IMS
D3	1.17	3.25	-	0.3	0.8	9.6	Fe(II) HS
Sext1	0.50	0	50.0	0.24	1.00	43.8	Fe_2O_3_1

Error for the CS and ΔE_Q determined by residual of the fitting in Fit model 2 is between 0.1 - 0.2 mm s^{-1}. Error for the H_0 in Fit model 2 is 0.5 – 1.4 T.

Table S4. Summary of SEC-MS parameters for FeNC electrodes measured at potentials of 0.9 V, 0.75 V, 0.6 V, 0.2 V, and a return to 0.9 V at room temperature using Fit model 2.

	CS [mm s^{-1}]	ΔE_Q [mm s^{-1}]	Lorentzian linewidth	Gaussian linewidth	Relative area %	Assignment
In situ 0.9 V						
D1	0.38	1.01	0.2	0.65	47.7	Fe^{III} HS
D2	0.35	2.50	0.3	0.8	22.3	Fe^{II} IMS
D3	1.03	3.10	0.3	0.8	19.0	Fe^{II} HS
In situ 0.75 V						
D1	0.42	1.01	0.2	0.65	39.3	Fe^{III} HS
D2	0.35	2.50	0.3	0.8	24.6	Fe^{II} IMS
D3	1.03	3.10	0.3	0.8	25.2	Fe^{II} HS
In situ 0.6 V						
D1	0.42	1.00	0.2	0.65	26.7	Fe^{III} HS
D2	0.35	2.50	0.3	0.8	24.5	Fe^{II} IMS
D3	1.03	3.10	0.3	0.8	31.8	Fe^{II} HS
In situ 0.2 V						
D1	0.42	0.95	0.2	0.65	20.6	Fe^{III} HS
D2	0.38	2.83	0.3	0.8	28.2	Fe^{II} IMS
D3	1.03	3.10	0.3	0.8	31.2	Fe^{II} HS
In situ 0.9 V_{back}						
D1	0.38	1.00	0.2	0.7	32.2	Fe^{III} HS
D2	0.35	2.50	0.3	0.8	26.6	Fe^{II} IMS
D3	1.03	2.95	0.3	0.8	21.2	Fe^{II} HS

Error for the CS and ΔE_Q determined by residual of the fitting in Fit model 2 is between 0.1 - 0.2 mm s^{-1}. Error for the H_0 in Fit model 2 is 0.5 – 1.4 T.

Table S5. Data for the bar chart plotted in Figure 17.

	Area %	Error %
E3E4		
Residual D1	18.7	1.0
E6E7		
Residual D1	20.4	1.3
E8E9		
Residual D1	22.1	2.0
E2E5		
Residual D1	18.2	2.8

Table S6. Data for the bar chart plotted in Figure 18.

	Area %	Error %
E3E4 0.9 V		
Residual D1	19	1.5
E6E7 0.75 V		
Residual D1	19	1.6
E8E9 0.6 V		
Residual D1	7	2.0
E2E5 0.2 V		
Residual D1	0	0

Reference:

(1) Heppe, N.; Gallenkamp, C.; Paul, S.; Segura-Salas, N.; von Rhein, N.; Kaiser, B.; Jaegermann, W.; Jafari, A.; Sergueev, I.; Krewald, V.; Kramm, U. I.Substituent Effects in Iron Porphyrin Catalysts for the Hydrogen Evolution Reaction. *Chemistry – A European Journal* **2023**, *29* (10), e202202465. DOI:10.1002/chem.202202465.

(2) Gridin, V.; Segura-Salas, N.; Saveleva, V. A.; Theis, P.; Haller, S.; Ribeiro, C. C.; Hofmann, K.; Stark, R.; Kramm, U. I.Enhancing the Stability of FeNC Catalysts in PEMFCs by Improved Purification. *American Chemical Society Catalysis* **2024**, *14* (14), 10951–10963. DOI:10.1021/acscatal.4c02930.

(3) Kramm, U. I.; Herrmann-Geppert, I.; Behrends, J.; Lips, K.; Fiechter, S.; Bogdanoff, P.On an Easy Way to Prepare Metal-Nitrogen Doped Carbon with Exclusive Presence of MeN4-Type Sites Active for the ORR. *Journal of the American Chemical Society* **2016**, *138* (2), 635–640. DOI:10.1021/jacs.5b11015.

(4) Stoll, S.; Schweiger, A.EasySpin, a Comprehensive Software Package for Spectral Simulation and Analysis in EPR. *Journal of Magnetic Resonance* **2006**, *178* (1), 42–55. DOI:10.1016/j.jmr.2005.08.013.

(5) Bera, A.; Bimmermann, S.; Gerschel, P.; Jyoti Barman, D.; Gerndt, L.; Lohmiller, T.; Abdiaziz, K.; Schnegg, A.; Orio, M.; H Hetterscheid, D. G.; Bren, K. L.; Roemelt, M.; Apfel, U.-P.; Ray, K.; Bera, A.; Barman, D. J.; Gerndt, L.; Lohmiller, T.; Römelt, M.; Ray, K.; Bimmermann, S.; Gerschel, P.; Apfel, U.; Orio, M.; H Hetterscheid, D. G.; Bren, K. L.Mechanistic Promiscuity in Cobalt Mediated CO2 Reduction Reaction: One- versus Two-Electron Reduction Process. *Angewandte Chemie International Edition* **2025**, e202503705. DOI:10.1002/anie.202503705.

(6) Bill, E.MF2, Version 2.7.3. Max Planck Institute for Chemical Energy Conversion.

(7) Rancourt, D. G.; Lagarec, K.Recoil User Manual -- Mossbauer Spectral Analysis Software for Windows. University of Ottawa 1998.

(8) Ni, L.; Gallenkamp, C.; Wagner, S.; Bill, E.; Krewald, V.; Kramm, U. I.Identification of the Catalytically Dominant Iron Environment in Iron- and Nitrogen-Doped Carbon Catalysts for the Oxygen Reduction Reaction. *Journal of the American Chemical Society* **2022**, *144* (37), 16827–16840. DOI:10.1021/jacs.2c04865.

(9) Ferretti, A. M.; Barra, A. L.; Forni, L.; Oliva, C.; Schweiger, A.; Ponti, A.Electron Paramagnetic Resonance Spectroscopy of Iron(III)-Doped MFI Zeolite. 1. Multifrequency CW-EPR. *Journal of Physical Chemistry B* **2004**, *108* (6), 1999–2005. DOI:10.1021/jp0370371.

(10) Kramm, U. I.; Ni, L.; Wagner, S.Fe Mössbauer Spectroscopy Characterization of Electrocatalysts. *Advanced Materials* **2019**, *31* (31), 1805623. DOI:10.1002/adma.201805623.

(11) Gallenkamp, C.; Kramm, U. I.; Proppe, J.; Krewald, V.Calibration of Computational Mössbauer Spectroscopy to Unravel Active Sites in FeNC Catalysts for the Oxygen Reduction Reaction. *International Journal of Quantum Chemistry* **2021**, *121* (3), e26394. DOI:10.1002/qua.26394.

(12) Li, J.; Sougrati, M. T.; Zitolo, A.; Ablett, J. M.; Oğuz, I. C.; Mineva, T.; Matanovic, I.; Atanassov, P.; Huang, Y.; Zenyuk, I.; Di Cicco, A.; Kumar, K.; Dubau, L.; Maillard, F.; Dražić, G.; Jaouen, F.Identification of Durable and Non-Durable FeNx Sites in Fe–N–C Materials for Proton Exchange Membrane Fuel Cells. *Nature Catalysis* **2020**, *4* (1), 10–19. DOI:10.1038/s41929-020-00545-2.

(13) Xu, X.; Zhang, X.; Kuang, Z.; Xia, Z.; Rykov, A. I.; Yu, S.; Wang, J.; Wang, S.; Sun, G.Investigation on the Demetallation of Fe-N-C for Oxygen Reduction Reaction: The Influence of Structure and Structural Evolution of Active Site. *Applied Catalysis B: Environmental* **2022**, *309*, 121290. DOI:10.1016/j.apcatb.2022.121290.

(14) Li, X.; Cao, C. S.; Hung, S. F.; Lu, Y. R.; Cai, W.; Rykov, A. I.; Miao, S.; Xi, S.; Yang, H.; Hu, Z.; Wang, J.; Zhao, J.; Alp, E. E.; Xu, W.; Chan, T. S.; Chen, H.; Xiong, Q.; Xiao, H.; Huang, Y.; Li, J.; Zhang, T.; Liu, B.Identification of the Electronic and Structural Dynamics of Catalytic Centers in Single-Fe-Atom Material. *Chem* **2020**, *6* (12), 3440–3454. DOI:10.1016/j.chempr.2020.10.027.

(15) Williams, K. F. E.; Johnson, C. E.Mossbauer Spectroscopy Measurement of Iron Oxidation States in ¨ Float Composition Silica Glasses. *Journal of Non-Crystalline Solids* **1998**, *226* (1–2), 19–23. DOI:10.1016/s0022-3093(98)00361-5.

(16) Dunaeva, E. S.; Uspenskaya, I. A.; Pokholok, K. V.; Minin, V. V.; Efimov, N. N.; Ugolkova, E. A.; Brunet, E.Coordination and RedOx Ratio of Iron in Sodium-silicate Glasses. *Journal of Non-Crystalline Solids* **2012**, *358* (23), 3089–3095. DOI:10.1016/j.jnoncrysol.2012.08.004.
